# Rapid Radiations Outweigh Reticulations During the Evolution of a 750-Million-Year-Old Lineage of Cyanobacteria

**DOI:** 10.1093/molbev/msaf244

**Published:** 2025-10-01

**Authors:** Carlos J Pardo-De la Hoz, Diane L Haughland, Darcie Thauvette, Sydney Toni, Spencer Goyette, William White, Ian D Medeiros, Luc Cornet, Petr Dvořák, Diego Garfias-Gallegos, Jolanta Miadlikowska, Nicolas Magain, François Lutzoni

**Affiliations:** Department of Biology, Duke University, Durham, NC 27708, USA; Alberta Biodiversity Monitoring Institute, University of Alberta, Edmonton, AB T6G 2E9, Canada; Department of Renewable Resources, Faculty of Agricultural, Life & Environmental Sciences, University of Alberta, Edmonton, AB T6G 2H1, Canada; Alberta Biodiversity Monitoring Institute, University of Alberta, Edmonton, AB T6G 2E9, Canada; Alberta Biodiversity Monitoring Institute, University of Alberta, Edmonton, AB T6G 2E9, Canada; Beaty Biodiversity Museum, University of British Columbia, Vancouver, BC V6T 1Z4, Canada; Department of Biological Sciences, Vanderbilt University, Nashville, TN 37235, USA; Department of Biology, Duke University, Durham, NC 27708, USA; Department of Botany, National Museum of Natural History, Smithsonian Institution, Washington, DC 20560, USA; BCCM/ULC, InBios Research Center, Université de Liège, Liège 4000, Belgium; Department of Botany, Faculty of Science, Palacký University Olomouc, Olomouc 783 71, Czechia; Department of Biology, Duke University, Durham, NC 27708, USA; Department of Biology, Duke University, Durham, NC 27708, USA; Evolution and Conservation Biology, InBioS Research Center, Université de Liège, Liège 4000, Belgium; Department of Biology, Duke University, Durham, NC 27708, USA

**Keywords:** ANI, average nucleotide identity, anomaly zone, bacterial species, cyanobacterium, lichens, *Nostoc*, Peltigerales, species delimitation, symbiosis

## Abstract

Species are a fundamental unit of biodiversity. Yet, the existence of clear species boundaries among bacteria has long been a subject of debate. Here, we studied species boundaries in the context of the phylogenetic history of *Nostoc*, a widespread genus of photoautotrophic and nitrogen-fixing cyanobacteria that includes many lineages that form symbiotic associations with plants (e.g. cycads and bryophytes) and fungi (e.g. cyanolichens). We found that the evolution of *Nostoc* was characterized by eight rapid radiations, many of which were associated with major events in the evolution of plants. In addition, incomplete lineage sorting associated with these rapid radiations outweighed reticulations during *Nostoc* evolution. We then show that the pattern of diversification of *Nostoc* shapes the distribution of average nucleotide identities (ANIs) into a complex mosaic, wherein some closely related clades are clearly isolated from each other by gaps in genomic similarity, while others form a continuum where genomic species boundaries are expected. Nevertheless, recently diverged *Nostoc* lineages often form cohesive clades that are maintained by within-clade gene flow. Boundaries to homologous recombination between these cohesive clades persist even when the potential for gene flow is high, i.e. when closely related clades of *Nostoc* co-occur or are locally found in symbiotic associations with the same lichen-forming fungal species. Our results demonstrate that rapid radiations are major contributors to the complex speciation history of *Nostoc.* This underscores the need to consider evolutionary information beyond thresholds of genomic similarity to delimit biologically meaningful units of biodiversity for bacteria.

## Introduction

Bacterial cells reproduce clonally but may exchange genetic material through horizontal gene transfers (HGT; Thomas and Nielsen 2005). As a result, their genomes often contain a mixture of loci inherited vertically and horizontally (Lawrence and Ochman 1998; Mostowy et al. 2017). These chimeric genomes have fueled two long-standing debates: whether bacterial evolution follows a bifurcating tree-like pattern (Doolittle 1999; Daubin 2002; Coleman et al. 2021), and whether bacterial species can be defined as distinct biological entities (Cohan 2002; Doolittle 2012; Shapiro et al. 2016).

The frequency of HGT varies depending on the mechanism of DNA integration and the relatedness of the donor and recipient genomes. Specifically, nonhomologous recombination can occur between distantly related genomes and typically involves accessory rather than core genes (Frost et al. 2005; Oliveira et al. 2017). In contrast, homologous recombination (HR) is more likely between closely related genomes and affects both accessory and core genes (Fraser et al. 2007; Everitt et al. 2014). There is growing evidence that HR in bacteria resembles gene flow in sexually reproducing eukaryotes, such that decreasing frequencies of HR between diverging genomes act as boundaries that fit the biological species concept (Bobay and Ochman 2017; Cobo-Simón et al. 2023).

Barriers to HR have been estimated to emerge at various levels of genome sequence identity, ranging from 90% to 98% ANI (Diop et al. 2022). This variation could be due to differences in the length of the nearly identical DNA fragments required to initiate HR, which varies across bacterial lineages (Shen and Huang 1986; Diop et al. 2022). These findings are in agreement with the common use of the 95% ANI threshold to delimit bacterial species based on genomic data (Konstantinidis and Tiedje 2005; Parks et al. 2020). In addition, a large-scale survey of prokaryotic genomes revealed a putative gap in the distribution of ANIs that spans 83% to 95% ANI, which has been interpreted as evidence of a universal species boundary (Jain et al. 2018; Rodriguez-R et al. 2021). However, the wide range of sequence identity levels associated with barriers to HR suggests that ANI boundaries could be centered around different sequence identities in different lineages. Therefore, we need to study the distribution of ANI gaps within a phylogenetic framework to assess the adequacy of ANI thresholds for bacterial species delimitation.

HR patterns can also drive a diversification process wherein species are cohesive recombining populations that diverge as barriers to HR arise (Shapiro et al. 2016; Stanojković et al. 2024). As part of this process, allele variation can be unlinked between loci due to recombination, which can lead to conflicts between phylogenies of different genes (Sakoparnig et al. 2021). One scenario where unlinked allele variation leads to conflicts among gene trees is due to incomplete lineage sorting (ILS), where ancestral polymorphisms are preserved through speciation events and the allele sorting differs from the primary history of population divergence (Figure S1a; Degnan and Rosenberg 2009). Conflicts among gene trees can also result from fragmented speciation, where genetic isolation is achieved asynchronously across the genome, leading to gene flow barriers at some loci while others continue to recombine freely (Figure S1b; Retchless and Lawrence 2010; Lawrence 2013). Conflicts due to ILS or fragmented speciation are more likely to occur when the time intervals between speciation events are short. For ILS, shorter intervals decrease the chance that any allele of the polymorphic loci will fixate before subsequent divergence (Figure S1a; Maddison 1997). Similarly, in fragmented speciation, shorter intervals decrease the probability that all loci will be isolated before the next divergence (Figure S1b; Lawrence 2013). Therefore, rapid radiations—characterized by successive speciation events occurring over a short timescale—can be a major source of phylogenetic conflicts among loci in bacteria.

Rapid radiations are expected to generate distinct patterns of phylogenetic conflict compared with reticulations, where genetic information is exchanged between distant, well-separated lineages (Figure S1). When ILS or fragmented speciation are the sources of phylogenetic conflict, the frequency of conflicts should increase as speciation intervals become shorter (Whitfield and Lockhart 2007; Lopes et al. 2021). Additionally, a greater proportion of the conflicting relationships may be recovered with weak statistical support as speciation intervals decrease due to fewer substitutions accumulating along short internal branches (Huang and Knowles 2009; Roycroft et al. 2020). In some rapid radiations, the patterns of phylogenetic conflict may fit the expectations of the anomaly zone: a region of tree parameter space where the most likely gene tree is discordant with the species tree (Degnan and Rosenberg 2006; Rosenberg 2013). Inferring species trees in the presence of anomaly zones resulting from rapid radiations is one of the main challenges of modern phylogenetics, especially for maximum likelihood inferences based on concatenated datasets (Kubatko and Degnan 2007; Mendes and Hahn 2018; Cloutier et al. 2019; Chafin et al. 2021; Morales-Briones et al. 2021; Pardo-De la Hoz et al. 2023). Nevertheless, rapid radiations have received little attention in phylogenetic studies of bacteria, where phylogenetic discordance is often regarded as a synonym of reticulated evolution (Murray et al. 2016; Martinez-Gutierrez and Aylward 2021).

In this study, we focus on *Nostoc*, a common and widespread genus of photoautotrophic and nitrogen-fixing cyanobacteria in the order Nostocales (Komárek et al. 2014; Dvořák et al. 2020). *Nostoc* often forms symbioses with plants (e.g. cycads and some bryophytes) and lichen-forming fungi (e.g. most cyanolichens of the order Peltigerales). In all these symbioses, *Nostoc* transfers fixed nitrogen to the plant and fungal symbionts (Warshan et al. 2018; Darnajoux et al. 2019). Consequently, *Nostoc* is recognized as a model to study plant and lichen symbiotic interactions, as well as biological nitrogen fixation (Magain et al. 2017; Warshan et al. 2018; Darnajoux et al. 2019). However, the scope of many studies is currently limited by the lack of meaningful and reliable units of biodiversity in this genus (Cornet et al. 2021). Here, we used 151 genomes and metagenome-assembled genomes (MAGs) to characterize genomic species boundaries in *Nostoc* within a phylogenomic framework and delimit such units. We first inferred a phylogenomic species tree with estimates of divergence times and quantified patterns of phylogenetic conflict to explore the contribution of ILS vs reticulations to *Nostoc* evolution. This enabled us to detect and date rapid radiations that occurred throughout the evolution of *Nostoc* and to identify successive speciation events that fit the expectations of the anomaly zone. Then, we surveyed the distribution of pairwise ANIs among *Nostoc* genomes to determine whether there is a uniform gap across the phylogeny of *Nostoc* that spans the expected 83% to 95% ANI range (Jain et al. 2018), which would be indicative of a homogeneous species boundary. We used these results, along with estimates of recent gene flow, to propose a classification scheme for *Nostoc* strains that integrates phylogenetic, genomic, and ecological information. Finally, we genotyped a collection of 2,316 lichenized *Nostoc* strains from a systematic regional-scale sampling to confirm that barriers to gene flow are maintained between closely related, co-occurring, species-level clades of *Nostoc* that we delineated in this study.

## Results and Discussion

### Rapid Radiations and ILS Were More Prevalent Than Reticulations During *Nostoc* Evolution

Our first goal was to infer the evolutionary history of *Nostoc.* We used a dataset of 151 genomes, including 124 newly generated *Nostoc* MAGs from cyanolichens sampled globally (Supplementary Data S1a). Of these new MAGs, 80 could not be assigned to a known species in the Genome Taxonomy Database (Supplementary Data S1a; Parks et al. 2022), demonstrating that the genomic diversity of *Nostoc* is highly underexplored.

**Fig. 1.**
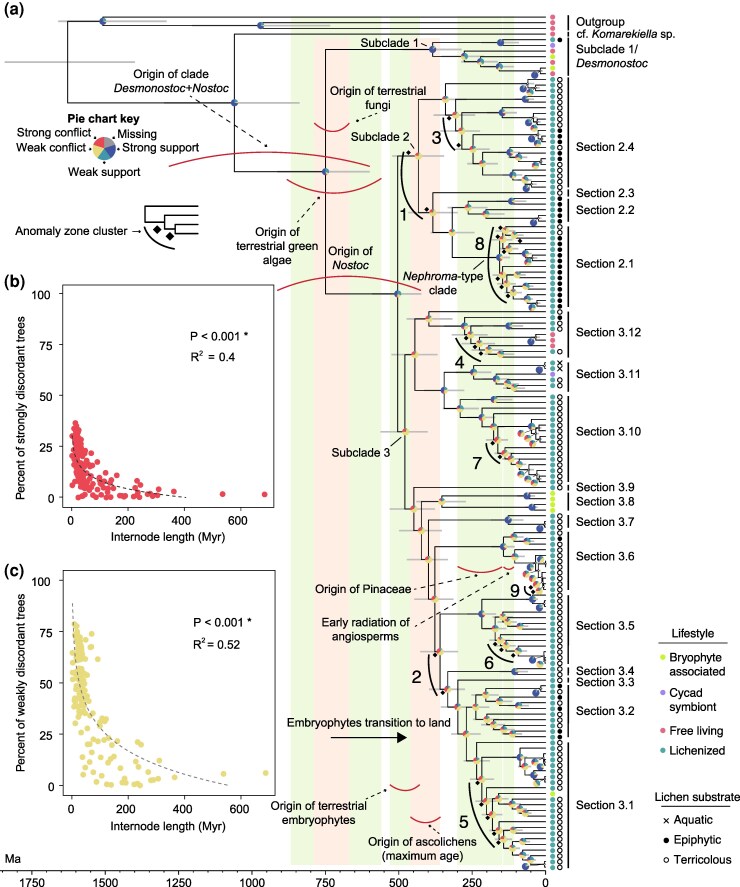
*Nostoc* phylogenetic history is characterized by multiple rapid radiations associated with plant evolution. a) Phylogenomic tree of *Nostoc* with estimates of divergence times including 151 taxa (Supplementary Data S1a). The topology was inferred with weighted-ASTRAL (Zhang and Mirarab 2022) using 1,519 gene trees. The gray node bars show the 95% highest posterior density of divergence times estimated with MCMCTree (dos Reis and Yang 2011). Pie charts show the proportion of the 1,519 gene trees that recovered each node with strong support, strong conflict, weak support, or weak conflict, or that were not scored due to missing data. We used 95% UFboot as the support threshold to assess conflicts. The delimitation of subclades 1 to 3 is partially based on Otálora et al. (2010), but both of our phylogenomic analyses (see Figure S2) recovered a different topology compared with their study, which was based solely on *rbcLX*. We also found that the 16S sequence of the type species of the genus *Demonostoc* falls within subclade 1. *Desmonostoc* was segregated from *Nostoc* and the two genera are sister (Hrouzek et al. 2013), thus, subclade 1 likely corresponds to *Desmonostoc*. The lineage labeled “cf. *Komarekiella* sp.” corresponds to strain *Nostoc* sp. B 2019, which is classified as *Nostoc* in GenBank but probably represents the closely related genus *Komarekiella* (Scotta Hentschke et al. 2017 ) according to GTDB. Terricolous cyanolichens include those growing directly on soil, mosses, and rocks. The vertical color strips, concave-up red arcs, and dashed arrows indicate estimated major evolutionary events for plants (green strips) and fungi (peach strips; Lutzoni et al. 2018). Concave-down red arcs indicate the estimated age of major events during *Nostoc* evolution. The early radiation of angiosperms includes the crown age of angiosperms until the crown age of Pentapetalae (Magallón et al. 2013). The maximum age for the origin of ascolichens corresponds to the stem age of the clade that includes Arthoniomycetes, Dothideomycetes, Eurotiomycetes, Lecanoromycetes, and Lichinomycetes (Díaz-Escandón et al. 2022). Numbered black arcs indicate anomaly zone clusters. b) and c) show the relationship between topological conflicts and time between speciation events. Each dot corresponds to an internal branch from the *Nostoc* species tree (a). The values on the *X* axis indicate the median branch length in millions of years, and the *Y* values are the percentage of gene trees that strongly (b) or weakly (c) reject each given internode. The dashed lines represent the predicted values from the linear model we fitted to the log-transformed data.

We then inferred a species tree with divergence time estimates and quantified phylogenetic conflicts by comparing the topology of each gene tree to the species tree (Fig. 1a and Figure S2a). We found that the number of phylogenetic conflicts was associated with the time elapsed between speciation events (Fig. 1b–c). More specifically, longer internodes (i.e. more time between speciation events) were associated with a higher proportion of congruent gene trees (Figure S3a). Conversely, shorter internodes (i.e. less time between speciation events) were associated with a higher proportion of both weakly and strongly supported conflicting gene trees (Fig. 1b–c). Importantly, the fraction of weakly supported conflicts was consistently larger than the fraction of strongly supported conflicts for short interval times (Fig. 1b–c). In addition, the proportion of congruent sites was also associated with internode lengths (Figure S3c). These findings strongly suggest that most phylogenetic conflicts resulted from rapid successive speciation events (Figure S1a–b; Whitfield and Lockhart 2007; Huang and Knowles 2009; Lawrence 2013; Roycroft et al. 2020; Lopes et al. 2021).

We also detected nine clusters of short consecutive internodes where phylogenetic conflicts fit the expectations of the anomaly zone (Fig. 1a). One of them (anomaly zone cluster 9; Fig. 1a) involves internodes within a species-level clade (phylogroup V; Figure S2a). The other eight correspond to interspecific divergences where node age estimates are largely overlapping (anomaly zone clusters 1 to 8; Fig. 1a). This indicates that they are part of rapid radiations and we will refer to them as such. All topological incongruences between coalescent and concatenated trees are associated with internode clusters that fit the expectations of the anomaly zone (Figure S2; Mendes and Hahn 2018; Cloutier et al. 2019). Moreover, the conflicting relationships have strong support in both trees, but they are local rearrangements of branches around internodes within an anomaly zone (Figure S2). These patterns are hallmarks of ILS associated with rapid radiations (Cloutier et al. 2019; Leducq et al. 2022; Pardo-De la Hoz et al. 2023).

Phylogenetic conflicts may also result from reticulations that are best represented by a network rather than a fully bifurcating tree (Huson 1998; Huson and Bryant 2006). We quantified the proportion of quartets from 1,519 gene trees that fit either a tree-like model, where conflicts are due to ILS, or a nontree like model, where conflicts are due to reticulations (Allman et al. 2019; Rhodes et al. 2021; Bjorner et al. 2023). We found that up to 73.8% of quartets in our phylogenomic dataset fit a tree-like ILS model (Table S1) compared with 26.2% fitting a nontree like reticulation model. This further supports that ILS is the main cause of phylogenetic conflicts in *Nostoc*.

**Fig. 2.**
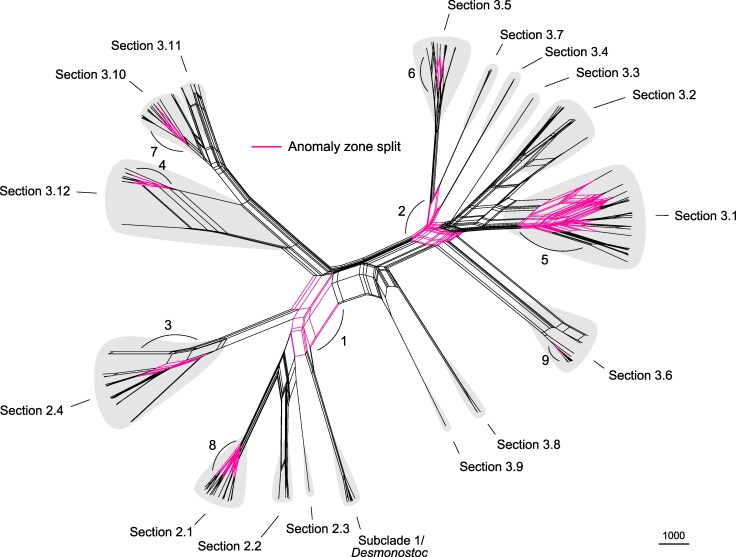
Reticulations are common between closely-related lineages of *Nostoc* during rapid diversification associated with anomaly zones. Phylogenetic split network inferred with NANUQ (Allman et al. 2019). Parallel edges are associated with the same split of taxa. The edge lengths represent split weights, which are proportional to the fraction of quartets supporting a given split. Numbered black arcs indicate areas of the network that correspond to the nine anomaly zone clusters shown in Fig. 1a.

We then used the results of this model-fitting analysis to infer a phylogenetic split network and found that several areas of complex reticulations correspond to relationships that fall in the anomaly zone (Fig. 2). These reticulations involve close rather than distant relatives and likely represent ongoing gene flow between rapidly diverging species during these radiations. Our results demonstrate that fully bifurcating trees do not capture the complexity of the speciation history in bacteria, especially for rapid radiations associated with anomaly zones (Fig. 2; Doolittle 1999; Pardo-De la Hoz et al. 2023). Nevertheless, the network recovered all major lineages delineated in the species tree (i.e. sections 2.1 to 2.4, sections 3.1 to 3.12, and subclade 1/*Desmonostoc*; Figs. 1a and 2). Moreover, most of them are subtended by long edges (Fig. 2), which indicates strong support for these relationships in the data (Allman et al. 2019). Therefore, our integration of species tree and network inferences yielded a robust phylogenomic framework while highlighting areas of complex speciation history linked to reticulations between close relatives and ILS resulting from rapid radiations in *Nostoc*.

### *Nostoc* Rapid Radiations are Associated With Major Events in Plant Evolution

We found that the crown age of the clade that includes *Desmonostoc* and *Nostoc*, ca. 750 (913 to 599) Ma, was contemporaneous with the estimated minimum age for the origin of terrestrial green algae (881 to 562 Ma) and terrestrial fungi (789 to 670 Ma; Fig. 1a; Lutzoni et al. 2018). This suggests that nitrogen fixation by terrestrial *Nostoc-*like cyanobacteria might have facilitated the transition to land by photoautotrophic green algae and heterotrophic fungi at a time when nitrogen and carbon were limited in terrestrial environments (Knack et al. 2015; Lenton and Daines 2017). Nevertheless, most of the diversification of *Nostoc* (starting 590 to 423 Ma) occurred after the origin of terrestrial embryophytes (530 to 430 Ma; Magallón et al. 2013; Lutzoni et al. 2018; Warshan et al. 2018; Servais et al. 2019), including all eight rapid radiations we detected (Fig. 1a). The association between land plant evolution and *Nostoc* diversification might be related to both the establishment of symbiotic associations and the availability of new terrestrial habitats with a diverse array of selective pressures (Lutzoni et al. 2018; Dahl and Arens 2020). There is fossil evidence that nonvascular plants occasionally harbored intercellular cyanobacterial symbionts ca. 400 Ma (Kidston and Lang 1921; Krings et al. 2009)—an association reminiscent of the present-day symbioses between *Nostoc* and some hornworts and liverworts (Villarreal Aguilar and Renzaglia 2006; Warshan et al. 2018; Nelson et al. 2019). However, the morphological features of the fossilized cyanobacteria suggest that they are closer relatives to the order Oscillatoriales than to *Nostoc* (Kidston and Lang 1921; Krings et al. 2009). Early *Nostoc* may have formed epiphytic rather than intercellular symbioses with early nonvascular plants similar to the interaction between extant *Nostoc* and some mosses (Warshan et al. 2017, 2018; Carrell et al. 2022).

Five of the eight rapid radiations (anomaly zone clusters 4 to 8 in Fig. 1a) occurred during a period (299 to 112 Ma) characterized by the origin and diversification of extant Pinaceae until the early radiation of flowering plants (Fig. 1a; Magallón et al. 2013; Lutzoni et al. 2018). In an early phylogenetic study of *Nostoc* using 16S rDNA sequences, Rikkinen et al. (2002) found two major clades and labeled them by their signature lichen mycobiont genus: (i) the *Peltigera*-type clade, which included *Nostoc* of terrestrial cyanolichens, free-living *Nostoc* strains, and a symbiotic *Nostoc* from the roots of a cycad; and (ii) the *Nephroma*-type clade, which only included *Nostoc* of epiphytic cyanolichens. The *Nephroma-*type clade corresponds to the lineage in anomaly zone cluster 8 in Fig. 1a, which we also found to be comprised mostly of *Nostoc* living in epiphytic cyanolichens growing on woody conifers and angiosperms. The cluster 8 radiation occurred contemporaneously with the origin and early radiation of flowering plants (Fig. 1a). Contrary to a previous suggestion (Nelsen et al. 2020), our results support an early origin of *Nostoc*, i.e. before the origin of ascolichens (Fig. 1a), and that the emergence of environments dominated by flowering plants was contemporaneous with the rapid diversification of most *Nostoc* that form associations with epiphytic cyanolichens.

The two *Nostoc* clades identified by Rikkinen et al. (2002) also prompted the popular hypothesis that cyanolichen communities form guilds structured by substrate (i.e. the epiphytic *Nephroma* guild and the terrestrial *Peltigera* guild) where *Nostoc* photobionts are shared within, but not among, those guilds (Rikkinen 2003; Fedrowitz et al. 2012; Dal Grande et al. 2014; Belinchón et al. 2015; Zúñiga et al. 2015; Zúñiga et al. 2017; Kaasalainen et al. 2021; Duran-Nebreda and Valverde 2023). However, our results confirm that *Nostoc-*like cyanobacteria found in lichens belong to at least three major lineages (subclade 1/*Desmonostoc*, and *Nostoc* subclades 2 and 3; Fig. 1a), all of which include strains with diverse lifestyles or associated with lichens from multiple substrates (Fig. 1a). Therefore, the two-guild model does not capture the evolutionary diversity of *Nostoc*, which implies that the mechanisms that underlie the interaction dynamics of *Nostoc* in cyanolichens probably involve a more complex combination of eco-evolutionary processes (Lu et al. 2018; Chagnon et al. 2019; Rolshausen et al. 2020; Pardo-De la Hoz et al. 2022; Rodríguez-Arribas et al. 2023).

### *Nostoc* Diversification Patterns Resulted in Heterogeneous Species Boundaries

**Fig. 3.**
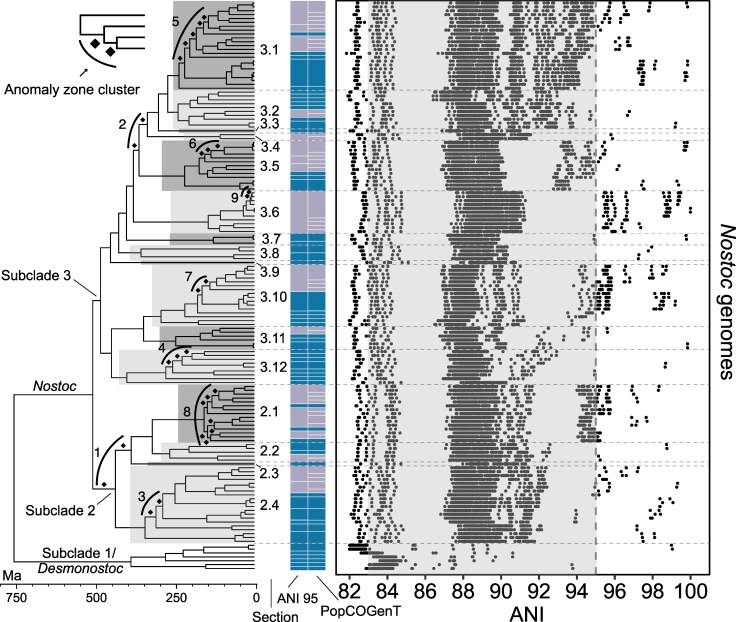
*Nostoc* diversification patterns resulted in heterogenous species boundaries. The dot plot shows the distribution of ANI values between all pairs of *Nostoc* and *Desmonostoc* genomes. Each row of dots shows the ANI values between a single *Nostoc* or *Desmonostoc* genome (at the tip of the corresponding terminal branch) and all the other *Nostoc* or *Desmonostoc* genomes included in this phylogeny. The vertical gray shade (spanning 83% to 95% ANI) shows the range that the ANI gap is expected to span (Jain et al. 2018). The vertical dashed line shows the 95% ANI threshold typically used for bacterial species delimitation (Konstantinidis and Tiedje 2005; Parks et al. 2020). The tree and branch lengths are the same as in Fig. 1a, but without the outgroup taxa and cf. *Komarekiella* sp. The numbers with decimal point to the right of the tree correspond to the 16 sections (highlighted with two different shades of gray) that we delimited within subclades 2 and 3. The two columns with color blocks show the genome clusters inferred using 95% ANI and PopCOGenT; blue indicates clusters supported by both methods, lilac indicates discordant clusters.

Our next goal was to explore genomic species boundaries within the phylogenetic framework of *Nostoc.* We calculated ANI between all pairs of *Nostoc* and *Desmonostoc* genomes available to us and found that the distribution of ANIs is more complex than expected if species boundaries (gaps) were homogenously distributed among lineages of these sister genera (Figs. 3 and S4). There is a gap centered around 86% ANI (Figs. 3 and S4) but this gap does not correspond to the expected universal species boundary spanning 83% to 95% ANI (Jain et al. 2018). Instead, we observed a mosaic of genomic continuity mixed with gaps spanning different ranges of ANI (Fig. 3). Importantly, the distribution of ANI values is largely structured by the diversification pattern of *Nostoc,* such that clades subtended by longer branches are separated from the rest by larger ANI gaps. For example, the *Nostoc* s. str. clade (i.e, subclades 2 and 3) is separated from sister subclade 1/*Desmonostoc* by a long branch (Fig. 3). Accordingly, the ANIs on the left side of the first gap (< ∼85% ANI) correspond to the distances between *Nostoc* and subclade 1/*Desmonostoc*, whereas the ANIs on the right side of the first gap (> ∼87% ANI) are mostly distances between genomes from subclades 2 and 3 (Fig. 3). The same is true for lineages within the *Nostoc* subclades, such as sections 3.5 and 3.6, which are both subtended by long branches and display additional ANI gaps closer to the expected 95% ANI boundary (Fig. 3). In contrast, *Nostoc* section 3.1 originated from a rapid radiation characterized by multiple consecutive short internodes. In that case, the ANIs form a continuum that spans roughly 88% to 95% ANI (Fig. 3).

Speciation has long been viewed as a continuum that is expected to generate heterogeneous rather than universal species boundaries (Drès and Mallet 2002; Kollár et al. 2022; Stanojković et al. 2024). Our results indicate that *Nostoc* is no exception, and that variation in diversification rates may underlie whether gaps in the distribution of ANIs are present or not. This implies that the recognition of biologically meaningful units of diversity in bacteria must go beyond genomic similarity thresholds and include a pluralistic approach that integrates multiple sources of evolutionary and ecological information (Palmer et al. 2019; Dvořák et al. 2023). Therefore, we propose a delimitation scheme for *Nostoc* that integrates those aspects.

We first delineated 16 sections within *Nostoc* subclades 2 and 3 (sections 2.1 to 2.4 and 3.1 to 3.12; Figs. 1–3) by considering the evolutionary isolation based on branch lengths, ANI clustering, and ecology of the strains. We then used this framework to validate and refine *Nostoc* phylogroups proposed in previous studies based on *rbcLX* sequences. We retrieved 1,098 public sequences of *rbcLX* from free-living and symbiotic *Nostoc* from previous phylogenetic studies (O’Brien et al. 2013; Magain et al. 2017; Chagnon et al. 2018; Magain et al. 2018; Miadlikowska et al. 2018; Pardo-De la Hoz et al. 2018; Supplementary Data S1b). Then, we placed them in our phylogenomic framework and sorted them by section to infer section-specific phylogenies. We found that 32 of the 43 previously delimited *Nostoc* phylogroups are monophyletic (Figure S5a–n). However, these clades were sometimes embedded within a set of less structured but closely related strains that had been assigned to multiple phylogroups (e.g. section 3.1, Figure S5a). In those cases, we considered the entire set to be a species complex. Species complexes are useful when boundaries are unclear, such as when radiations resulted in a near-continuum of genomic diversity (e.g. section 3.1 in Figs. 3 and S5a). We provide guidelines for the classification of new *Nostoc* strains into our scheme using either genomic or single-locus data in the GitHub repository for this study: https://github.com/cjpardodelahoz/nostoc.

### *Nostoc* Phylogroups Remain Distinct When Cooccurring With Closest Relatives

The *Nostoc* phylogroups reflect phylogenetic structure within the 16 sections we delimited here at a global scale (Figure S5). However, in lineages with wide geographic distribution, phylogenetic structure might be detected spuriously due to biased sampling on distant ends of a genomic continuum (Chambers and Hillis 2020; Chambers et al. 2023), such as the ones we found in some *Nostoc* lineages (e.g. section 3.1 in Figs. 3 and S5a). Therefore, our final goal was to use a systematic spatial sampling to determine whether the phylogroups we delimited were robust in cases with high potential for gene flow with their closest relatives (i.e. frequent spatial cooccurrence and sharing of fungal symbiotic partners).

**Fig. 4.**
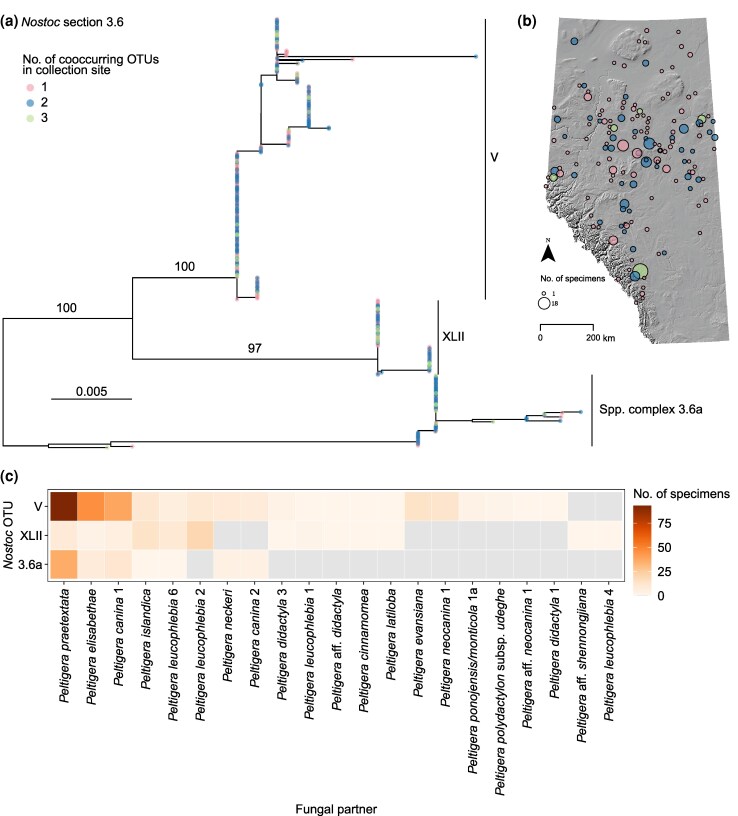
*Nostoc* lineage boundaries are maintained despite cooccurrence and shared fungal symbiotic partners (*Peltigera*). This example is from *Nostoc* section 3.6 (Figures 1–3) from cyanolichens collected in Alberta, Canada. We considered each labeled clade as one OTU (i.e. phylogroups V and XLII, and spp. complex 3.6a) for a total of three OTUs. a) Maximum likelihood tree of 426 *Nostoc rbcLX* sequences. The color of the circles at the branch tips indicates the number of cooccurring *Nostoc* OTUs from section 3.6 at the specific sites where each specimen was collected. Numbers above branches are UFBoot2 support values. Branch lengths represent the expected number of nucleotide substitutions per site. b) Relief map of Alberta showing the distribution of 152 sites where the lichenized *Nostoc* were collected. c) Interaction matrix between *Nostoc* and *Peltigera* lichen-forming fungal partners. Each cell in the matrix shows the frequency of the respective combination of *Nostoc* OTU and its fungal partner *Peltigera* in Alberta.

We genotyped lichenized *Nostoc* strains associated with 2,316 cyanolichen specimens collected systematically by the Alberta Biodiversity Monitoring Institute (ABMI) across 366 sites in the province of Alberta, Canada. We sequenced the *rbcLX* region and classified them with the scheme we described above. We also clustered the *Nostoc* genomes using both 95% ANI and rates of recent gene flow using PopCOGenT (Fig. 3; Arevalo et al. 2019). We found that most of the strains from Alberta (1,996; Supplementary Data S1c and d) belong to section 2.4 (214), section 3.1 (1,183), section 3.5 (173) and section 3.6 (426). In all four sections, we found that the phylogroups we identified were robust even when they co-occurred with closely related *Nostoc* from the same section (Figs. 4a–b and S6a–c). This suggests that factors other than geographic isolation play an important role in preventing gene flow among these OTUs (i.e. phylogroups or species complexes) and maintaining genetic differentiation (Cadillo-Quiroz et al. 2012; Stanojković et al. 2024). Populations may be structured along fine-scale heterogeneity that drives differential adaptations, as has been shown for Archaea and other Bacteria (Shapiro et al. 2012; Chase et al. 2019; Wang et al. 2020).

Another potential driver of genetic isolation among *Nostoc* lineages is divergent selection resulting from specialization on different symbiotic fungal partners. We genotyped the fungal partners of the lichenized *Nostoc* strains from Alberta and instead found that, at the regional scale, the OTUs from a given *Nostoc* section often share fungal symbiotic partners in a nested manner (Figs. 4c and S6a–c). Phylogroups III (section 2.4) and VIId (section 3.5) were exceptions to these trends because their interactions are with fungal partners that are rarely or never found with other *Nostoc* OTUs from the same section (Figure S6b, c). This reciprocal specificity could underlie the genetic divergence of these *Nostoc* phylogroups from the other lineages in their sections.

When different *Nostoc* OTUs associate with the same lichen-forming species at a regional scale (e.g. sections 3.1 and 3.6; Figs. 4c and S6a), genetic differentiation between *Nostoc* populations can still emerge if they associate with different lichen-forming fungal partners at local scales. In that case, partner sharing would be less frequent between co-occurring *Nostoc* strains compared with *Nostoc* strains from different sites. Instead, we found that partner sharing is equally or more frequent between pairs of co-occurring *Nostoc* strains than between pairs of *Nostoc* strains from different sites in Alberta (Table 1, Supplementary Data S1f–h). The difference is more pronounced for strain pairs that belong to the same section (Table 1, Supplementary Data S1f–h), which is likely driven by frequent asexual reproduction of lichen thalli, resulting in the vertical transmission of *Nostoc* at local scales. This indicates that symbiotic specialization is probably not the main driver of genetic differentiation in *Nostoc* symbionts of cyanolichens.

**Table 1.** Summary of fungal partner sharing between cooccurring and noncooccurring pairs of cyanolichen specimens with Nostoc OTUs from section 3.6 in Alberta, Canada (Fig. 4a)

Nostoc oTUs	Total specimen pairs	Cooccurring specimen pairs	Noncooccurring specimen pairs	Cooccurring specimen pairs sharing fungal partner	Noncooccurring specimen pairs sharing fungal partner	% of cooccurring specimen pairs sharing fungal partner	% of noncooccurring specimen pairs sharing fungal partner
V and XLII	18,078	107	17,971	26	1,561	24.29	8.68
3.6a and XLII	4,554	24	4,530	2	430	8.33	9.49
3.6a and V	17,292	169	17,123	50	4,207	29.58	24.56
XLII and XLII	2,346	35	2,311	9	367	25.71	15.88
V and V	34,191	499	33,692	120	6,251	24.04	18.55
3.6a and 3.6a	2,145	31	2,114	18	718	58.06	33.96

Overall, our findings show that OTU boundaries between *Nostoc* symbionts of cyanolichens are robust even when there is a high potential for gene flow between close relatives (i.e. frequent cooccurrence and found in association with the same *Peltigera* species). Nevertheless, the processes underlying the maintenance of gene flow boundaries may differ in nonlichenized *Nostoc* lineages. This is because the bacterial lifestyle can shape gene flow dynamics and natural selection, leading to alternative divergence mechanisms (e.g. environmental vs. human gut populations of *Escherichia coli*; Luo et al. 2011). Our phylogenomic framework should aid the discovery of these potential alternatives as more data becomes available for *Nostoc* with different lifestyles.

Our results also show that relevant units of biodiversity may be finer than ANI-delimited species. For example, *Nostoc* section 3.6 corresponds to one ANI cluster, but five different gene flow clusters (Fig. 3). More specifically, sister *Nostoc* phylogroups V and XLII (section 3.6) are part of the same ANI cluster (ANI-8), but they are in different gene flow clusters (PopCO-1 and PopCO-85, bolded taxa in Figure S5e). Phylogroup V is broadly distributed across multiple continents, whereas XLII has a circumboreal distribution (Figure S5e) (Magain et al. 2018). Both *Nostoc* phylogroups share fungal partners, but phylogroup V has a much broader partner range both globally (Figure S5e) and in Alberta (Fig. 4c). These genetic, geographic, and symbiotic differences imply that these phylogroups are neither evolutionary nor ecologically interchangeable, which is a fundamental property of biologically meaningful units of biodiversity (Cohan 2019).

Our findings underscore the importance of assessing bacterial biodiversity in the context of their evolutionary history. We showed that a fully bifurcating tree does not capture the complexity of the evolutionary history of *Nostoc* (Fig. 2). However, most of the complexity results from rapid radiations and ILS rather than reticulations between distantly related lineages (Figs. 1–2, S2, and Table S1). Additionally, different diversification patterns can result in both clearly distinct lineages separated by gaps in genomic similarity as well as lineages with a continuum of genomic diversity (Fig. 3). Nevertheless, recently diverged lineages (e.g. *Nostoc* phylogroups) that display genomic cohesion are common even when they cooccur with close relatives and share symbiotic partners (Figs. 4 and S6, Table 1, Supplementary Data S1f–h). These are more meaningful biodiversity units to track when studying phenomena such as the maintenance of barriers to gene flow and the evolution of symbiotic interactions in cyanobacteria.

## Methods

### Sampling and Sequencing of *Nostoc* Genomes

We used a total of 151 genomes that represent three major lineages of *Nostoc-*like cyanobacteria (Magain et al. 2017, 2018). This included 24 publicly available genome assemblies (Warshan et al. 2017; Zhu et al. 2017; Gagunashvili and Andrésson 2018; Warshan et al. 2018; Halsør et al. 2019; Shang et al. 2019; Bell-Doyon et al. 2020; Chen et al. 2021) and 124 newly generated MAGs of *Nostoc* strains associated with 17 genera of cyanolichen-forming fungi (Supplementary Data S1a). We also included the genomes of *Anabaena cylindrica* PCC 7122 (Shih et al. 2013), *Aphanizomenon flos-aquae* NIES 81 (Cao et al. 2014), and *Cylindrospermum stagnale* PCC 7417 (Shih et al. 2013) to use as outgroup taxa for the phylogenetic analyses (Supplementary Data S1a). One of the public genomes classified as *Nostoc* in NCBI (*Nostoc* sp. B 2019; Supplementary Data S1a) is classified as *Komarekiella* sp. in the Genome Taxonomy Database.

For the newly generated MAGs, we extracted metagenomic DNA from the lobe tips of healthy and clean lichen thalli using a 2% SDS lysis followed by phenol:chloroform separation, isopropanol precipitation, and ethanol cleanup (full protocol in Appendix S1). Then, metagenomic libraries (150 bp paired end) were prepared with the KAPA HyperPrep kit (Roche Sequencing Solutions, Pleasanton, CA, USA) following manufacturer's instructions and sequenced on three Illumina NovaSeq 6000 S Prime flow cells. Library preparation and sequencing were conducted by the Duke Sequencing and Genomic Technologies core facility.

### Metagenomic Assembly, Binning, and Curation

We first examined read quality and adapter content using FastQC v0.11.17. Then, we trimmed low-quality bases (PHRED < Q20) and adapters using Trimmomatic v0.39. We only used paired reads that were >75 bp after trimming for subsequent analyses. We assembled the trimmed reads using SPAdes v3.14.1 (Bankevich et al. 2012; Nurk et al. 2017) with the –meta option and with kmer lengths 55, 75, and 95. To quantify the depth of coverage of the assembled contigs for binning, we mapped the SPAdes-corrected reads to the metagenomic assembly using Bowtie v.2.3.5.1 (Langmead and Salzberg 2012) and samtools v1.9 (Danecek et al. 2021), and then extracted a summary of the depth of coverage per contig using the jgi_summarize_bam_contig_depths script from MetaBAT2 (Kang et al. 2019). The assembled contigs and their depths were used as input for initial binning with MetaBAT2.

To identify the cyanobacterial bins obtained from each metagenomic library, we used the lineage-specific workflow from CheckM v1.1.7 (Parks et al. 2015). CheckM places the genome bins onto a bacterial reference genome tree and selects lineage-specific markers to calculate genome quality metrics. We used the output of CheckM to identify the genome bins that belonged to Cyanobacteria. We obtained a single cyanobacterial genome bin from 118 of our metagenomic libraries. In five of our metagenomic libraries (P2083, P2170, P10246, P10247, and P12560), we found more than one cyanobacterial genome bin. We then conducted a preliminary phylogenetic analysis to determine which of the cyanobacterial genome bins belonged to *Nostoc*. For this, we used 37 publicly available genomes from *Nostoc* (Supplementary Data S1a) that were included in a recent study on Nostocales phylogenomics (Pardo-De la Hoz et al. 2023) as a reference. For the reference genomes and all cyanobacterial genomes from the metagenomic libraries, we ran BUSCO v4.1.3 (Simão et al. 2015) using the “cyanobacteria_odb10” as the reference database (Kriventseva et al. 2019). This database consists of 773 single-copy orthologs conserved across Cyanobacteria. We aligned the nucleotide sequences of the 773 BUSCO markers using MAFFT v7.475 (Katoh and Standley 2013) and PAL2NAL v14 (Suyama et al. 2006) as described in the section below. We trimmed all sites with gaps and generated a concatenated alignment that we used to infer a maximum likelihood tree in IQ-Tree v1.6.12 (Nguyen et al. 2015) with a GTR + G model and 1000 UFBoot2 replicates. With the resulting tree, we identified genome bins from four libraries (P2083, P2170, P10247 and P12560) that fell outside of *Nostoc* and excluded them from all subsequent analyses. All of those four libraries contained an additional cyanobacterial bin that fell within *Nostoc* and we used those for subsequent analyses.

We refined the *Nostoc* genome bins with information from the assembly graph produced by SPAdes to achieve the highest quality MAGs. First, we used Graphbin2 (Mallawaarachchi et al. 2020), a binning refinement program that applies a label propagation algorithm to improve the binning results from other tools. Then, we used Bandage (Wick et al. 2015) to visualize the metagenomic assembly graphs and labeled the contigs (graph edges) that were included in the *Nostoc* genome bins by both MetBAT2 and Graphbin2. This allowed us to remove contaminant, chimeric, and duplicated contigs, as well as include *Nostoc* contigs that were not binned by MetaBAT2 because they were either too small (<2,500 bp) or had aberrant coverage (e.g. repetitive and mobile genetic elements, and rRNA genes). The refinement was partly possible because the *Nostoc* genome was typically an isolated component in the metagenome assembly graphs, and the *Nostoc* contigs had very high depth of coverage (median 175×; Supplementary Data S1a) compared with the rest of the metagenome. The manual refinement was done using the anvi-refine interactive interface from Anvio v7.1 (Eren et al. 2015). We obtained 124 *Nostoc* MAGs with 98% median BUSCO completeness (using the nostocales_odb10 database; Supplementary Data S1a), all of which included a full copy of the 16S rRNA gene.

We then used Anvio v7.1 (Eren et al. 2015) to search for single-nucleotide variants (SNVs) in the *Nostoc* MAGs to detect potential strain heterogeneity. We found that the median number of SNVs per MAG was 5,394 (max: 105,836; Figure S7a). This represents less than 0.1% of the average MAG size (∼7.4 million bps, Supplementary Data S1a). Moreover, the median number of SNVs per genome that fell within one of the 1,517 BUSCO markers we used for phylogenetic analyses was 214 (max: 36,834; Figure S7b), or <0.02% of the length of the concatenated alignment of those markers (i.e. 1,547,142 sites, 642,002 of which are parsimony-informative). We also found that, for most MAGs, the median proportion of reads that differ from the consensus base was <0.1 (Figure S8e; see Figure S8a–j for a summary of the full distributions). The same was true for SNVs within BUSCO markers (Figure S8f). Importantly, the proportion of reads that differ from the consensus base does not scale with the total number of SNVs in the genome (Figure S7c) or within BUSCO markers (Figure S7d). This means that the departure from the consensus base remained low even for the few MAGs with a relatively high number of SNVs (Figure S7c–d).

Overall, the SNV analyses indicate that significant allele variation is both rare (< 0.1% of MAG size on average; Figure S7a–b) and highly skewed toward the consensus (average fraction of deviating alleles is <0.1 per SNV for most MAGs; Figure S8e–f). This variation is consistent with the notion that cyanolichen thalli contain a dominant strain of *Nostoc* along with a low abundance of closely related strains of the same species-level clade (i.e. within 99.9% ANI on average).

We also classified the contigs from the *Nostoc* MAGs into chromosome and plasmid origin using PlasX (Yu et al. 2024) and Deeplasmid (Andreopoulos et al. 2022). We combined the classification outputs from both tools to obtain a consensus. PlasX has higher accuracy and scalability than Deeplasmid (Yu et al. 2024). Therefore, if a contig was classified as plasmid only by Deeplasmid, we only considered it if the contig depth deviated by >20× from the median coverage of the chromosome contigs as classified by PlasX.

### Phylogenetic Inference

We first aligned the amino acid sequences of the 1,899 genes from the nostocales_odb10 database used by BUSCO using MAFFT v7.475 (Katoh and Standley 2013) with the –globalpair algorithm with 1,000 refinement iterations. We then obtained nucleotide alignments by back-translating the amino acid alignments using PAL2NAL v14 (Suyama et al. 2006) and the unaligned nucleotide sequences as input. Ambiguously aligned regions were removed by trimming all sites with gaps using trimAl v1.2rev59 (Capella-Gutierrez et al. 2009). We only kept the alignments of the 1,517 genes that had >200 variable sites and >136 taxa (i.e. >90%). In addition, we extracted the 16S rRNA gene and the *trnL* intron sequence from the genomes and aligned them with MAFFT as described above. These two markers will provide a link between our phylogenomic framework and many previous studies that characterized the molecular diversity of *Nostoc* using 16S or *trnL* sequences (Rajaniemi et al. 2005; Kaasalainen et al. 2015; Strunecký et al. 2023). A tutorial with examples of these links is available in the GitHub repository for this study: https://github.com/cjpardodelahoz/nostoc.

To infer gene trees, we first partitioned the coding nucleotide alignments into 1st, 2nd, and 3rd codon position and searched for the best partition scheme and substitution models using ModelFinder (Kalyaanamoorthy et al. 2017) and PartitionFinder2 (Lanfear et al. 2016) as implemented in IQ-Tree v1.6.12 (-m MFP + MERGE option; Nguyen et al. 2015). Then, we searched for maximum likelihood gene trees in IQ-Tree with 1000 UFBoot2 (Hoang et al. 2018) replicates. We used the resulting gene trees to infer a species tree with weighted-ASTRAL, which uses branch support values (i.e. UFBoot2) to generate weighting schemes for the quartet-based species tree inference to account for uncertainty in gene tree estimation (Zhang and Mirarab 2022). We also inferred a maximum likelihood tree with a concatenated alignment of the nucleotide sequences of the 1,517 BUSCO genes, the 16S rRNA gene, and the *trnL* intron. The substitution model selection and tree search were done using the same parameters as we did for the gene trees above. Overall, our phylogenetic analyses resulted in 1,519 single-locus trees, one weighted-ASTRAL species tree (Figs. 1a and S2a), and one maximum likelihood concatenated species tree (Figure S2b).

### Quantification of Phylogenetic Conflict

To quantify phylogenetic conflicts, we compared each of the 1,519 single-locus trees to the weighted-ASTRAL species tree. We used DiscoVista (Sayyari et al. 2018) to calculate the proportion of gene trees that strongly support, strongly reject, weakly support, and weakly reject each of the bipartitions in the weighted-ASTRAL tree. We used 95% UFBoot2 as the threshold to evaluate strong support. When a gene tree had missing taxa, the corresponding missing taxa were removed from the weighted-ASTRAL tree before evaluating conflict. Then, we used custom R functions and the R package *ggtree* v3.6.2 (Yu et al. 2017) to plot the results of the conflict analyses as pie charts on the weighted-ASTRAL tree. In addition, we compared the topologies of the weighted-ASTRAL and the concatenated tree to identify highly supported conflicts (Figure S2) using the Phylo.io interactive web server (Robinson et al. 2016).

We tested whether there was a relationship between time elapsed between speciation events (i.e. branch lengths in absolute time units) and the percentage of gene trees that strongly support, strongly reject, weakly support, and weakly reject each bipartition in the ASTRAL tree. We fitted four linear models between each of the percentage variables and the logarithm of branch lengths, e.g. percent with strong support ∼ log(branch length). Each model included 148 data points corresponding to the 148 internal branches of the ASTRAL tree. We used the lm() function in the *stats* package in R v4.2.2 (R Core Team 2013). The lm() function fits a linear model and tests the null hypothesis that the slope of the linear equation is equal to 0. We assessed significance with *α* = 0.01.

We also tested whether there was a relationship between the time elapsed between speciation events and the percentage of parsimony-informative sites that support each bipartition [i.e. site concordance factors (sCFs)] in the ASTRAL tree. To estimate sCFs, we first obtained maximum likelihood estimates of branch lengths in substitutions per site for the ASTRAL topology with the concatenated alignment of 1,519 loci described above. Then, we used the resulting tree and branch lengths, as well as the concatenated alignment, to estimate sCFs. Both analyses were performed in IQ-Tree (Mo et al. 2023). We fitted a linear model of the form sCF ∼ log(branch length) as described above.

To ensure that the patterns of phylogenetic conflict we observed are independent of the trace levels of strain heterogeneity we observed in the *Nostoc* MAGs, we also repeated the phylogenetic inferences and the quantification of phylogenetic conflicts after filtering genes with SNVs. For each MAG, we identified BUSCO genes with at least one SNV where the nonconsensus allele had a frequency ≥0.1 and removed those sequences from the single-locus alignments. We also removed six outlier taxa that had SNVs in more than 20% of their BUSCO genes. This filtered dataset contained 3.27% missing data compared with 0.97% missing data in the original dataset. The analyses based on the filtered dataset recapitulated all patterns from the original dataset (Figure S9a–d). We recovered a species tree topology identical to the tree in Fig. 1a (excluding the six outlier taxa) and with the same anomaly zone clusters. In addition, we found again that the proportion of both weakly and strongly supported phylogenetic conflicts, as well as the proportion of discordant sites, is associated with the time elapsed between speciation events (Figure S9a–d). This demonstrates that phylogenetic conflicts are not the result of strain heterogeneity or chimeric assemblies.

### Detection of Internodes in the Anomaly Zone

Equation 4 in (Degnan and Rosenberg 2006) can be used to calculate the value of *a*(*x*), which is the boundary of the anomaly zone for a branch of length *x* that has a descendant branch of length *y*. If *y* < *a*(*x*), then *x* and *y* are in the anomaly zone. To detect branches that fall in the anomaly zone in the *Nostoc* phylogeny, we calculated *a*(*x*) for each branch length *x* in the weighted-ASTRAL species tree and then compared *a*(*x*) to the length *y* of each descendant internal branch in coalescent units.

### Detection of Reticulations and ILS Using Species Network inference

We used the R package MSCquartets v1.1.2 (Rhodes et al. 2021) to test the fit of the multispecies coalescent model (MSC) to the distribution of quartet topologies from the 1,519 gene trees that we inferred. For each quartet, MSCquartets tests the null hypotheses that the quartet count concordance factors arose from a species quartet tree of unspecified topology (“T3”) under the MSC, which implies that the observed gene tree conflicts are due to ILS. The alternative hypothesis is that the quartet is not tree-like, which may be evidence for reticulations or the result of noise from gene tree error. Some of the reticulations inferred by MSCquartets may be equivalent to sustained gene flow between diverging lineages as in the fragmented speciation model (Figure S1b). This is difficult to ascertain with coalescent models because they assume that speciation is instantaneous (Retchless and Lawrence 2010). We then used the results of these tests to infer a species network splits graph under the Network MSC with the NANUQ algorithm (Allman et al. 2019). We set *β* = 0.1 and *α* = 1e−6. As recommended by Allman et al. (2019), we chose a small *α* given the high proportion of weakly supported conflicts in our dataset (Fig. 1a and c), which indicates a high prevalence of noise from gene tree error. However, we also report the results of the quartet tests with *α* = 1e−2, 1e−3, and 1e−5 (Table S1). We visualized the splits graph using SplitsTree v4.19.2 (Huson 1998).

### Divergence Time Estimation

There are no fossils that can be reliably assigned to *Nostoc*. Therefore, to infer divergence times for *Nostoc*, we first dated a phylogeny of the order Nostocales using fossils and geological calibrations. Then, we used several of the estimated age distributions within Nostocales as secondary calibrations to infer divergence times within *Nostoc*. For the Nostocales analysis, we used the 55 cyanobacterial taxa included in subset 0 of Pardo-De la Hoz et al. (2023; Supplementary Data S2a). We also included the genome of *Nostoc* sp. cyanobiont of *Peltigera malacea* JL33 (Supplementary Data S2a) so the split between *Nostoc* subclades 2 and 3 would be represented in the dated tree (Cornet et al. 2021; Pardo-De la Hoz et al. 2023). We inferred divergence times with MCMCTree, which allows Bayesian estimation of divergence times for a fixed topology and large phylogenomic alignments (Yang 2007; dos Reis and Yang 2011). We used the same topology as in Pardo-De la Hoz et al (2023; Supplementary Data S2a) and a concatenated amino acid alignment of the 1,648 BUSCO genes used in that study. To date the tree, we used two calibrations: (i) a maximum age for the root set to 2,700 Ma with default right tail probability *p_R_* = 0.025, which is based on geological evidence for the early origin of oxygenic photosynthesis (Farquhar et al. 2011; Uyeda et al. 2016); and (ii) a calibration for the crown age of Nostocales with a minimum age set to 1,600 Ma based on fossil evidence of akinete-like structures which have a single origin in Nostocales, and a maximum age set to 2,320 Ma, which is the lower bound for the rise in atmospheric oxygen and must have predated the evolution of heterocysts (Bekker et al. 2004; Tomitani et al. 2006). We used LG + G4 as the substitution model, an uncorrelated relaxed clock model with default priors, and a birth (*λ*)-death (*μ*) prior on node ages with *λ* = *μ* = 1 and sampling fraction *ρ* = 0.1. We sampled from both the prior and posterior distribution of divergence times using three MCMC chains with 100,000,000 generations, sampling every 1,000th generation, and discarded the first 20,000,000 generations as burnin. We assessed convergence by comparing the mean posterior node ages inferred with each of the three chains and checking that the effective sample size was >200.

For the divergence time estimation within *Nostoc*, we used MCMCTree with the topology of the weighted-ASTRAL tree and a concatenated alignment of the nucleotide sequences of the 1,519 loci dataset. We dated the tree with six secondary calibrations (i.e. 95% highest posterior density intervals) obtained from the dated Nostocales tree: (i) the root age was set between 1,160 and 1,840 Ma; (ii) the age of the outgroup clade, which was set between 990 and 1,620 Ma; (iii) the age of the most recent common ancestor of cf. *Komarekiella* sp. (*Nostoc* sp. B 2019) and subclades 1 to 3 was set between 710 and 1,410 Ma; (iv) the crown age of the clade that includes subclades 1 to 3 was set between 440 and 950 Ma; (v) the crown age of subclade 1/*Desmonostoc* was set between 150 and 460 Ma; and (vi) the age of the most recent common ancestor of *Nostoc* (i.e. subclades 2 and 3) was set between 180 and 590 Ma. All secondary calibration priors had a uniform distribution with soft bounds and tail probabilities *p_R_* = *p_L_* = 0.025, which allowed estimated ages to be outside the calibration range with a total probability density of 0.05. We used HKY + G5 as the substitution model, and the same clock and tree priors as for the Nostocales analysis. We sampled from both the prior and posterior distribution of divergence times using three MCMC chains with 14,000,000 generations, sampling every 1,000th generation, and discarded the first 4,000,000 generations as burnin. Convergence was assessed the same way as for the Nostocales analysis. The dated *Nostoc* tree with age estimates in newick format can be found in Supplementary Data S2b.

### Genome Clustering

We used FastANI v1.31 (Jain et al. 2018) to calculate the average nucleotide identity (ANI) and alignment fraction between every pair of *Nostoc* genomes in our sampling. We then used a custom R script to group the genomes into clusters with a threshold of 95% ANI (Goris et al. 2007; Jain et al. 2018; Olm et al. 2020). We also used PopCOGenT (Arevalo et al. 2019) to delimit clusters of genomes based on estimates of recent gene flow. Finally, we classified our genomes using the Genome Taxonomy Database Toolkit v.2.3.2 (Chaumeil et al. 2020) with the –skip_ani_screen flag. For both analyses, we only used the chromosome contigs from each MAG.

### Sequencing of Cyanolichens from Alberta

We used 2,316 cyanolichen specimens collected in 366 sites of 1 ha each by the Alberta Biodiversity Monitoring Institute (ABMI, www.abmi.ca; Supplementary Data S1c and d). The ABMI systematically surveys biodiversity in sites located in a 20-km grid across the province of Alberta, Canada. We genotyped the *Nostoc* photobionts and main fungal symbionts of the cyanolichen specimens using amplicon sequencing on PacBio SMRT Cells (Armanhi et al. 2016; Nelson et al. 2021). To do this, we extracted metagenomic DNA using the same protocol as for the generation of *Nostoc* MAGs (Appendix S1). Then, we amplified the *rbcLX* region of the *Nostoc* photobionts using primers CW and CX (Rudi et al. 1998), and the nrITS-partial LSU region from the fungal partners using primers ITS1F and LR3 (Vilgalys and Hester 1990; Gardes and Bruns 1993). We added tags at the 5′ end of these primers (5′-CTGGAGCACGAGGACACTGA-3′ to forward primers and 5′-GCTGTCAACGATACGCTACG-3′ to reverse primers) that allowed the attachment of sample-specific barcodes to the *rbcLX* and nrITS-partial LSU amplicons in a second PCR reaction. We used 384 barcodes (Supplementary Data S3) and used the same barcode sequence on both ends of each amplicon. Barcoded amplicons were pooled in sets of 384 samples and size selection was performed to remove fragments <700 bp using Mag-Bind TotalPure NGS (Omega Bio-tek) magnetic beads. The libraries were prepared and sequenced at the Duke Sequencing and Genomic Technologies core, with each pool sequenced in a separate PacBio SMRT Cell. We used PURC (Rothfels et al. 2017) to demultiplex the PacBio Circular Consensus Sequences and only kept sequences with >20× read depth.

### Classification of *Nostoc rbcLX* Sequences

We assembled a dataset that included (i) the full *rbcL* and *rbcX* sequences from the 151 reference taxa in our phylogenomic analyses (Supplementary Data S1a); (ii) the 2,316 *rbcLX* sequences from the ABMI cyanolichen specimens (Supplementary Data S1a); and (iii) 1,098 public *rbcLX* sequences that had been included in previous phylogenetic analyses of *Nostoc* that identified multiple phylogroups within the genus (Supplementary Data S1b; O’Brien et al. 2013; Magain et al. 2017; Chagnon et al. 2018; Magain et al. 2018; Miadlikowska et al. 2018; Pardo-De la Hoz et al. 2018). This last set consists mostly of *Nostoc* sequences from cyanolichens collected worldwide, but it also includes sequences from free-living and plant-symbiotic strains (Supplementary Data S1b). Initially, we retrieved all sequences included in the analyses of those previous studies and later removed 291 sequences that only spanned the *rbcX* region or were missing most of the 3′ end of the *rbcL* gene. We then aligned all 3,274 sequences using MAFFT with the –retree 1 and –maxiterate 0 flags and manually refined and excluded ambiguous regions and the spacer in Mesquite v3.70 (http://www.mesquiteproject.org/). We used this alignment to place the ABMI (ii) and public (iii) *rbcLX* sequences on the phylogenomic tree of *Nostoc* using the Evolutionary Placement Algorithm implemented in RAxML v8.2.12 (Berger et al. 2011; Stamatakis 2014) and the weighted-ASTRAL *Nostoc* tree (Fig. 1 and Figure S2a) as the reference topology. The EPA placed 98% (3,058) of the queries within one of the sections and subclades delimited in Figs. 1–3. We used the placements to sort the reference and query *rbcLX* sequences into 16 sets, one for each of the 16 sections in *Nostoc* subclades 2 and 3. Those sets of sequences were then aligned with MAFFT and refined manually in Mesquite. Sorting the sequences by section allowed the inclusion of the spacer region in the subsequent phylogenetic analyses of all alignments. We then inferred maximum likelihood trees from each alignment using IQ-Tree with 1000 UFBoot2 replicates.

We used the resulting trees to test the delimitations of 43 phylogroups that had been defined in previous phylogenetic studies of *Nostoc* based solely on *rbcLX* (O’Brien et al. 2013; Magain et al. 2017; Magain et al. 2018). For this, we removed the ABMI taxa from the trees and only examined the relationships among the public *rbcLX* sequences and the *rbcLX* sequences from the genomes included in the phylogenomic analyses (Figure S5a–n). This allowed us to determine the cases where the sequences previously assigned to a phylogroup were recovered as monophyletic and how these clades relate to the clusters delimited with genomic data (Fig. 3). We propose the recognition of 43 *Nostoc* phylogroups within *Nostoc* subclades 2 and 3 (32 delimited previously and 11 defined here; Table S2; Supplementary Data S1b; Figure S5a–n) that can be identified using genomic or *rbcLX* sequence data. Twenty-one of these phylogroups correspond to a single gene-flow cluster identified with PopCOGenT (Figure S5a–b, d–j, and n). In two cases, we merged a pair of sister clades that had been delimited as two phylogroups into one because they corresponded to the same gene-flow unit inferred by PopCOGenT (phylogroups XVI and XVIII, and phylogroups XIII and XLIII; Figure S5i). However, several of these phylogroups were recovered as clades nested within a set of less structured but closely related strains (e.g. section 3.1; Figure S5a). This is probably the result of rapid diversification leaving behind a near-continuum of diversity as we observed at broader phylogenetic scales in *Nostoc* (Fig. 3) and as evidenced by the presence of multiple internodes that fall in the anomaly zone within the sections (Fig. 3). Therefore, we consider these larger clades as species complexes. Altogether, our approach allowed us to classify the public *rbcLX* sequences into phylogroups and/or species complexes, sections, and subclades (Supplementary Data S1b). Finally, we used these delimitations to classify the ABMI sequences according to their position in the section trees relative to the public sequences. Altogether, 1,705 ABMI sequences were classified to phylogroup level; 2,307 were classified at least to section and species complex; and only 7 sequences have an uncertain position within the *Nostoc* tree (Supplementary Data S1c). These *incertae sedis* strains should be targets for future genome sequencing because they probably represent additional sections or subclades.

### Classification of Mycobionts From Alberta Cyanolichens

We examined all cyanolichen specimens and assigned preliminary identifications to the lichen-forming fungus (mycobiont) based on morphological traits (Supplementary Data S1c). Of the 2,316 cyanolichen specimens we used, 2,060 were from the lichen-forming fungal genus *Peltigera.* For those specimens, we assigned molecular species identifications by placing the nrITS and partial LSU sequences into the *Peltigera* phylogeny available on the T-BAS platform (https://guide-tbas.cifr.ncsu.edu/tbas) using the EPA algorithm (Carbone et al. 2017; Carbone et al. 2019). We also performed BLASTn searches of the nrITS sequences against a custom database that included all *Peltigera* sequences from previous studies on the phylogeny, systematics, and species delimitation within this genus (O’Brien et al. 2013; Miadlikowska et al. 2014; Magain et al. 2017; Chagnon et al. 2018; Magain et al. 2018; Miadlikowska et al. 2018; Pardo-De la Hoz et al. 2018). For the remaining cyanolichen specimens from other genera, we assigned molecular identifications at the genus or species-level based on BLAST searches of the nrITS sequences against the NCBI nucleotide database. Overall, we assigned molecular identifications to the lichen-forming fungus for 2,146 cyanolichen specimens (Supplementary Data S1c).

## Supplementary Material

msaf244_Supplementary_Data

## Data Availability

All sequence data were deposited in GenBank under BioProject accession PRJNA1066398. Amplicon sequence data is available under GenBank accessions KIFN01000001–KIFN01002316 (*rbcLX*), KIFO01000001–KIFO01002145 (nrITS), and KIFP01000001–KIFO01001677 (partial nrLSU). Metagenomic reads are available from the Sequence Read Archive under accessions SRR28386200–SRR28386311. *Nostoc* genome assemblies’ accession numbers are listed in Supplementary Data S1a. All the outputs from computational analyses and processing pipelines were deposited in the Dryad Digital Repository DOI: 10.5061/dryad.dv41ns25x. Phylogenomic and *rbcLX* trees and alignments of *Nostoc* are available on the T-BAS (https://guide-tbas.cifr.ncsu.edu/tbas) platform for download and placement of unknown *Nostoc* sequences. A tutorial for placement of unknown queries can be found in the GitHub repository for this study: https://github.com/cjpardodelahoz/nostoc/blob/main/tbas_tutorial/README.md. All code used to analyze the data in the present study can be found in the GitHub repository for this study: https://github.com/cjpardodelahoz/nostoc.
