# Supplementary material for: Rapid Radiations Outweigh Reticulations During the Evolution of a 750-Million-Year-Old Lineage of Cyanobacteria
Source: Mol Biol Evol. 2025 Oct 1;42(10):msaf244. doi: 10.1093/molbev/msaf244 (PMC12559999; doi:10.1093/molbev/msaf244)
Supplement: msaf244_Supplementary_Data [file msaf244_supplementary_data.zip › Supplementary Materials.docx]

**Additional Supplementary Materials**

**Supplementary Data 1.** Metadata and data summaries for the collections analyzed in this study. **a** Metadata for the 151 genomes used in the study. For the lichenized, bryophyte-associated, and cycad symbiont strains, the taxon name column indicates the name of the fungal or plant partner of each strain. Site IDs are provided for genomes from lichen specimens collected as part of systematic sampling efforts. Metadata for the sites is provided in **d** for Alberta Biodiversity Monitoring Institute (ABMI) sites and **e** for GoLife sites. **b** Table with the original and revised clade assignments (i.e., subclade, section, species complex, and phylogroup) for the 1,098 public *rbcLX* sequences of *Nostoc* that we re-classified as part of this study. **c** Table with clade assignments for the 2,316 *rbcLX* sequences from lichenized *Nostoc* collected by the ABMI in Alberta, Canada. **d** Metadata for the subset of ABMI sites from which the cyanolichens analyzed in this study were collected. The public coordinates point to a location that is within 5.5 km of the actual site center. Exact site coordinates remain confidential per ABMI policy. **e** Metadata for the GoLife sites where a subset of the cyanolichens were collected. **f–h** Summary of fungal partner sharing between cooccurring and non-cooccurring pairs of ABMI cyanolichen specimens with *Nostoc* OTUs from sections 3.1 **(f),** 3.5 (**g**), and 2.4 (**h**).

**Supplementary Data 2.** Dated trees supporting Fig. 1. **a** Dated tree of Nostocales in newick format, including the 95% highest posterior density of node ages. **b** Dated tree of *Nostoc* in newick format, including the 95% highest posterior density of node ages. This is the tree file used to make Figs. 1 and 3.

**Supplementary Data 3**. Barcode sequences used for dual indexing of *rbcLX* and ITS-partial nrLSU amplicons in order to pool them for PacBio sequencing. The barcodes are organized in sets of 96 in the input format for bulk ordering of 96-well plates of oligos from Integrated DNA Technologies, Inc.
