# Supplementary material for: Rapid Radiations Outweigh Reticulations During the Evolution of a 750-Million-Year-Old Lineage of Cyanobacteria
Source: Mol Biol Evol. 2025 Oct 1;42(10):msaf244. doi: 10.1093/molbev/msaf244 (PMC12559999; doi:10.1093/molbev/msaf244)
Supplement: msaf244_Supplementary_Data [file msaf244_supplementary_data.zip › Supplementary_materials.pdf]

**Appendix S1. DNA isolation protocol**

***Required supplies and equipment***

- 1.7 mL microcentrifuge tubes.
- 1.0 mm diameter Zirconia/Silica beads.
- Liquid nitrogen.
- Mini-Beadbeater (BioSpec Bioproducts) or similar tissue grinder.
- 2% SDS buffer (see recipe below).
- Phenol:chloroform:isoamyl alcohol 25:24:1.
- Chloroform:isoamyl alcohol 24:1.
- Isopropanol
- 70% ethanol.
- Sterile water.

***2% SDS buffer recipe***

Mix the following solutions at the specified volumes for a total volume of 50 mL:

| **Solution** | **Volume** |
|---|---|
| 5 M NaCl | 1.5 mL |
| 1 M Tris (pH 8) | 2.5 mL |
| 0.5 M NaEDTA | 1.0 mL |
| 10% (m/v) SDS | 10 mL |
| Sterile water | 35 mL |

***Procedure***

1. Place a piece of ~1 $cm^2$ of cyanolichen tissue into a 1.7 mL microcentrifuge tube.
2. Add Zirconia/Silica beads (1.0 mm diameter) until they cover at least half of the lichen tissue in the tube.
3. Freeze the tissue by submerging the tube in liquid nitrogen and quickly load it onto a Mini-Beadbeater and grind for at least 1 minute.
4. Add 500 µL of 2% SDS buffer into the tube. Invert the tube gently to ensure the powder is suspended in the buffer. Leave the tube overnight at room temperature.
5. Add 500 µL phenol:chloroform:isoamyl alcohol 25:24:1. Mix gently and leave for 5 minutes.

6. Centrifuge for 5 minutes at 12,000 rpm.
7. Transfer supernatant to a new 1.7 mL microcentrifuge tube. Use tips, do not pour out. Expect ~300 µL.
8. Add 250 µL of chloroform:isoamyl alcohol 24:1.
9. Centrifuge for 5 minutes at max speed.
10. Transfer supernatant to a new 1.7 mL microcentrifuge tube. Use tips, do not pour out. Expect ~200 µL.
11. Add 0.56x volume of cold (-20 C) isopropanol. Invert gently 2–3 times and centrifuge at 14,000 rpm for 2 minutes. Do not keep the isopropanol for long in the tube.
12. Remove supernatant carefully. Pour out but do not lose the pellet.
13. Add 150 µL of 70% ethanol. If there is a pigment in the pellet, wait for 5 minutes after adding the ethanol.
14. Centrifuge for 5 minutes at max speed.
15. Remove ethanol (pour out) and set tube upside down over a paper towel to let the ethanol dry for. Wait for at least 4 hours.
16. Resuspend the dry pellet in 50 µL of sterile water and store at -20 C.

**Supplementary Table 1.** Percentage of tree-like quartets for different clades and values of α for the hypothesis test implemented in MSCquartets. This can only be calculated for clades with $\geq$ 4 taxa.

| | Percentage of tree-like quartets | | | |
|---|---|---|---|---|
| **Clade** | **α = 1e-2** | **α = 1e-3** | **α = 1e-5** | **α = 1e-6** |
| Full network | 52.71 | 60.59 | 70.14 | 73.80 |
| Section 2.1 | 61.25 | 71.94 | 80.73 | 83.08 |
| Section 2.2 | 80 | 100 | 100 | 100 |
| Section 2.3 | - | - | - | - |
| Section 2.4 | 68.63 | 74.57 | 81.61 | 83.47 |
| Section 3.1 | 67.70 | 74.59 | 83.55 | 86.14 |
| Section 3.2 | 65.24 | 72.86 | 90.48 | 92.86 |
| Section 3.3 | - | - | - | - |
| Section 3.4 | - | - | - | - |
| Section 3.5 | 63.50 | 70.77 | 81.54 | 84.34 |
| Section 3.6 | 83.94 | 94.24 | 95.76 | 95.76 |
| Section 3.7 | - | - | - | - |
| Section 3.8 | 100 | 100 | 100 | 100 |
| Section 3.9 | - | - | - | - |
| Section 3.10 | 62.53 | 69.23 | 76.04 | 77.97 |
| Section 3.11 | 60.00 | 60.00 | 73.33 | 73.33 |
| Section 3.12 | 56.35 | 58.73 | 69.05 | 75.40 |

**Supplementary Table 2.** Summary of phylogenomic and *rbcLX* data supporting the delineations of 43 phylogroups validated or defined in the study. Monophyly was only evaluated when we had data available for > 1 strain. A cluster (ANI 95% or gene flow [PopCOGenT]) was exclusive if it only included strains from the given phylogroup. Cluster IDs are summarized in Supplementary Data 1a.

| Phylogroup | No. of genomes | Phylogenomic monophyly | No. of 95% ANI clusters | ANI 95% clusters exclusive ? | No. of gene flow clusters | Gene flow clusters exclusive ? | *rbcLX* monophyly |
|---|---|---|---|---|---|---|---|
| III | 3 | Yes | 1 | Yes | 1 | Yes | Yes |
| IVa | 2 | Yes | 1 | Yes | 1 | Yes | Yes |
| IVb | 4 | Yes | 2 | Yes | 3 | Yes | Yes |
| IVc | 3 | Yes | 1 | Yes | 1 | Yes | Yes |
| V | 7 | Yes | 1 | No | 1 | Yes | Yes |
| VI | 2 | Yes | 1 | Yes | 2 | Yes | Yes |
| VIIa | 3 | Yes | 1 | No | 1 | Yes | Yes |
| VIIb | 3 | Yes | 1 | Yes | 1 | Yes | Yes |
| VIIc | 3 | No | 3 | No | 3 | Yes | Yes |
| VIId | 0 | NA | NA | NA | NA | NA | Yes |
| VIIIa | 0 | NA | NA | NA | NA | NA | Yes |
| VIIIb | 0 | NA | NA | NA | NA | NA | Yes |
| IX | 0 | NA | NA | NA | NA | NA | Yes |
| Xa | 1 | NA | 1 | Yes | 1 | Yes | Yes |
| Xb | 0 | NA | NA | NA | NA | NA | Yes |
| XIa | 2 | Yes | 1 | Yes | 1 | Yes | Yes |
| Xib | 1 | NA | 1 | Yes | 1 | Yes | Yes |
| XIII.XLIII | 5 | Yes | 1 | Yes | 1 | Yes | Yes |
| XIV | 0 | NA | NA | NA | NA | NA | Yes |
| XV | 0 | NA | NA | NA | NA | NA | Yes |
| XVIa | 1 | NA | 1 | No | 1 | Yes | Yes |
| XVII | 0 | NA | NA | NA | NA | NA | Yes |
| XVI.XVIII | 4 | Yes | 1 | No | 1 | Yes | Yes |
| XIX | 1 | NA | 1 | Yes | 1 | Yes | Yes |
| XX | 1 | NA | 1 | No | 1 | Yes | Yes |
| XXI | 1 | NA | 1 | Yes | 1 | Yes | Yes |
| XXII | 1 | NA | 1 | Yes | 1 | Yes | Yes |

| | | | | | | | |
|---|---|---|---|---|---|---|---|
| XXIII | 1 | NA | 1 | Yes | 1 | Yes | Yes |
| XXV | 1 | NA | 1 | No | 1 | Yes | Yes |
| XXVII | 0 | NA | NA | NA | NA | NA | Yes |
| XXIX | 1 | NA | 1 | No | 1 | Yes | Yes |
| XXXIII | 7 | Yes | 1 | Yes | 1 | Yes | Yes |
| XXXIV | 1 | NA | 1 | Yes | 1 | Yes | Yes |
| XXXVII | 1 | NA | 1 | Yes | 1 | Yes | Yes |
| XXXVIII | 0 | NA | NA | NA | NA | NA | Yes |
| XL | 0 | NA | NA | NA | NA | NA | Yes |
| XLII | 1 | NA | 1 | No | 1 | Yes | Yes |
| XLIV | 2 | Yes | 1 | Yes | 1 | Yes | Yes |
| XLV | 1 | NA | 1 | Yes | 1 | Yes | Yes |
| XLVI | 1 | NA | 1 | No | 1 | Yes | Yes |
| XLVII | 2 | Yes | 1 | Yes | 1 | Yes | Yes |
| XLVIII | 2 | Yes | 1 | Yes | 1 | Yes | Yes |
| XLIX | 2 | Yes | 1 | Yes | 1 | Yes | Yes |

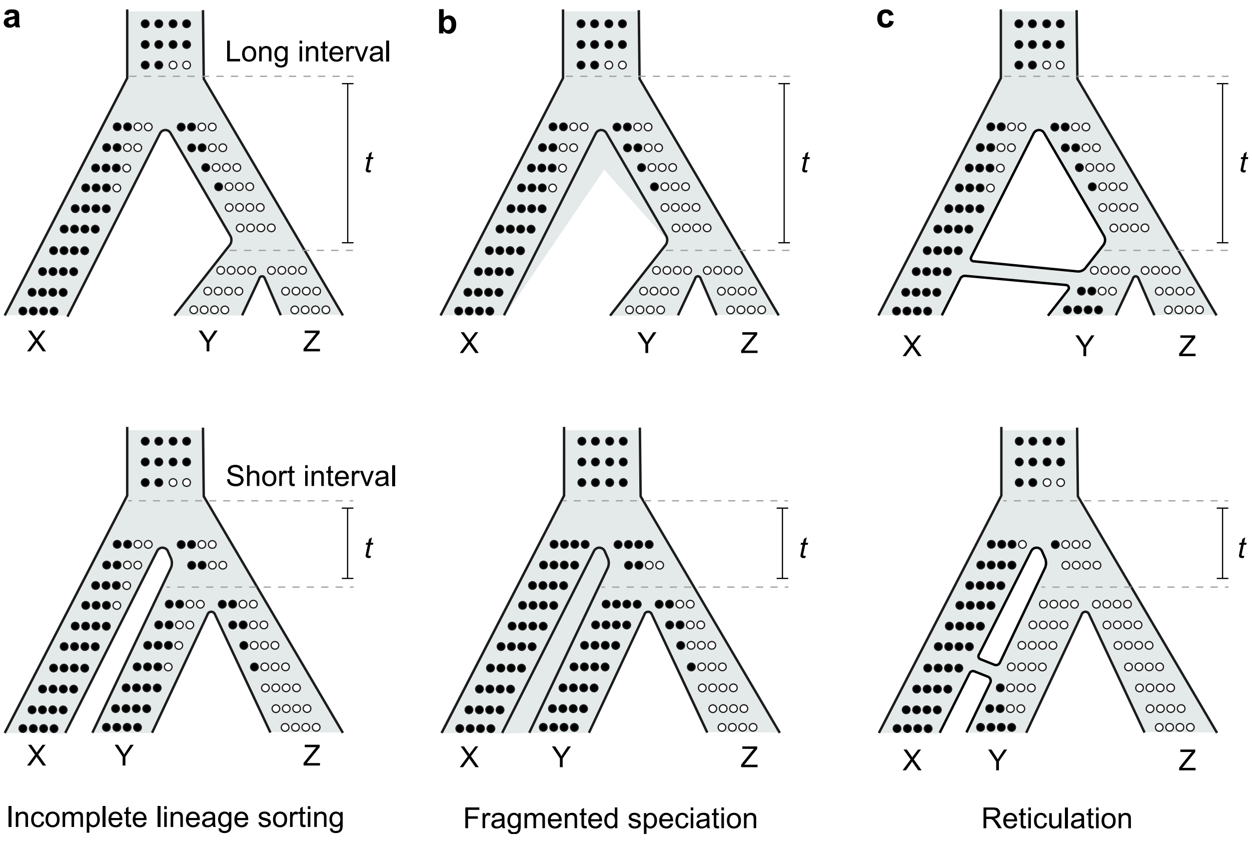


**Supplementary Figure 1.** Potential causes of conflicts among gene trees in bacteria and the effect of short time intervals between speciation events. **a** Incomplete lineage sorting. **b** Fragmented speciation. **c** Reticulated evolution. Black pipes depict speciation history. Gray shades show HR patterns for a focal locus. Dashed lines indicate speciation events. Closed and open circles represent alleles of a polymorphic locus. *t:* time interval between speciation events.

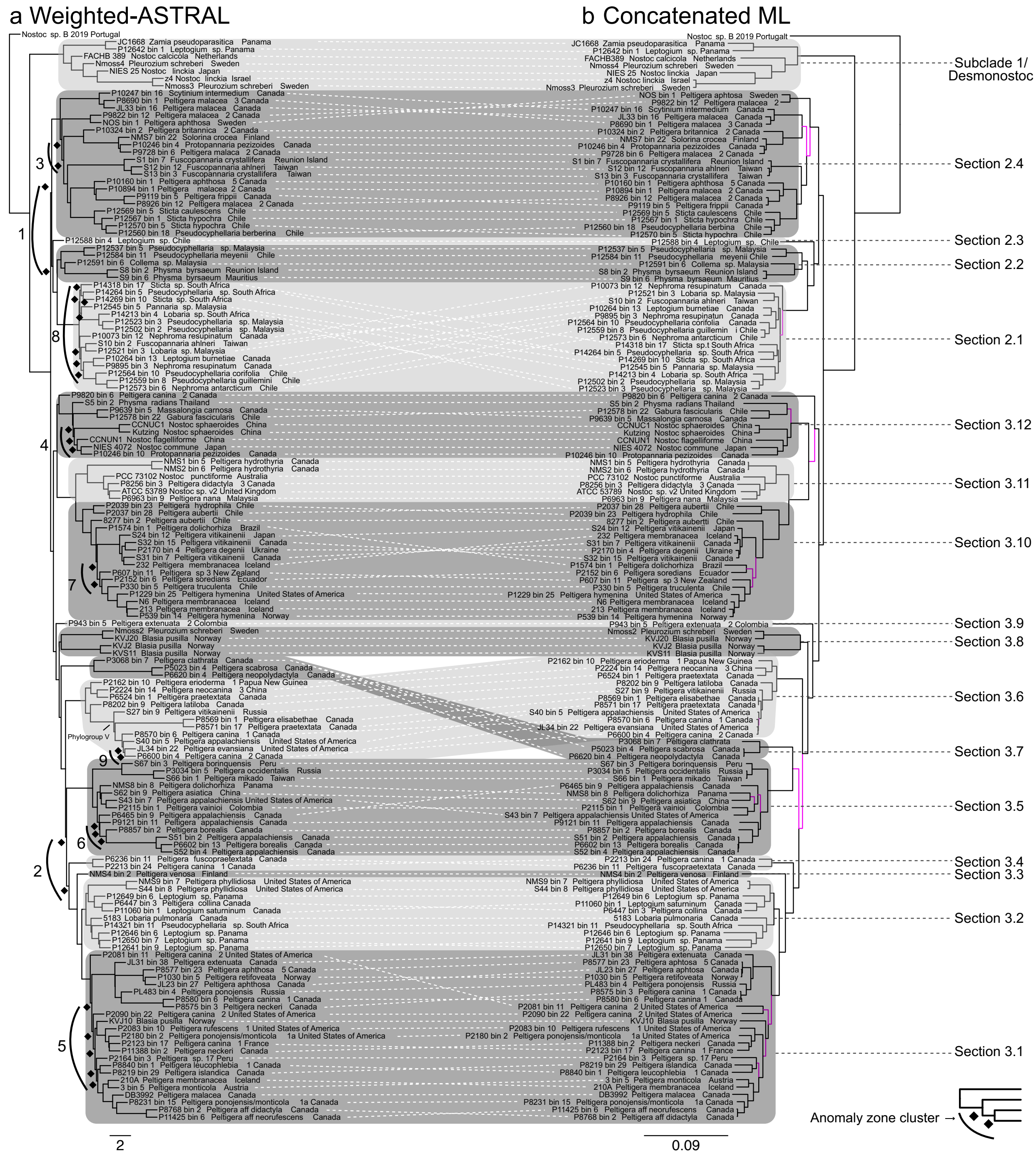


**Supplementary Figure 2.** Conflicts between coalescent and concatenated maximum likelihood trees are associated with anomaly zones. **a** Weighted-ASTRAL species tree of *Nostoc* genomes inferred from 1,519 gene trees. This is the same topology as in Figs. 1a and 3. Anomaly zone clusters correspond to those highlighted in Fig. 1a. Phylogroup V was delimited following the tree in Supplementary Fig. 5e. Branch lengths represent coalescent units. All branches have local posterior probability > 0.95. **b**

Maximum likelihood tree inferred from a concatenated matrix of the same 1,519 loci used to infer the tree in panel A. Fuchsia branches highlight topological bipartitions that are in strong conflict with the weighted-ASTRAL tree. Branch lengths represent expected nucleotide substitutions per site. All internodes have UFBoot2 > 95%. In both trees, the gray boxes highlight the sections delimited in Figs. 1–3. The strain *Nostoc* sp. B 2019 from Portugal probably represents the closely related genus *Komarekiella* according to GTDB.

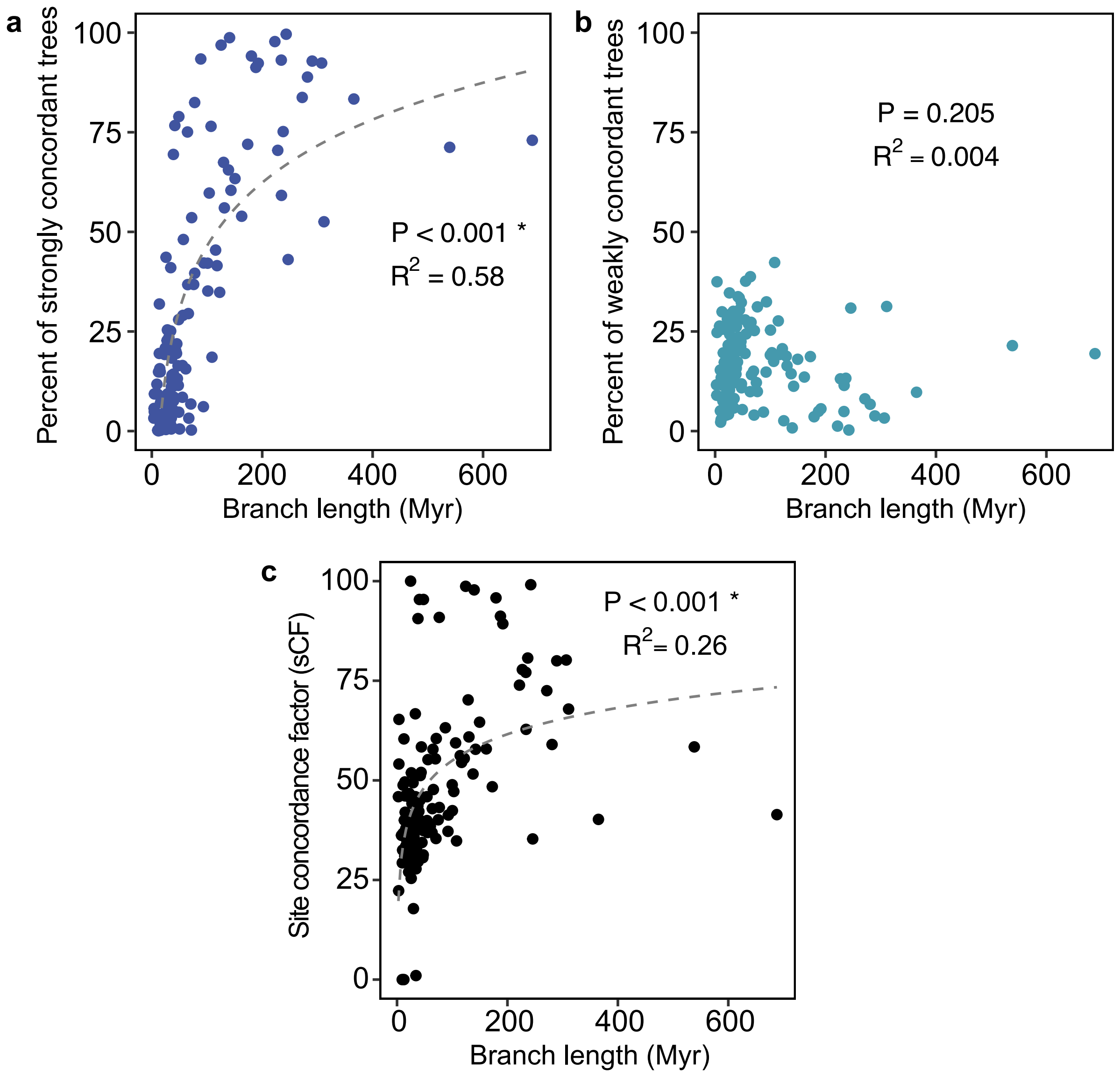


**Supplementary Figure 3.** Relationship between phylogenetic congruence and time between speciation events (branch lengths). Each dot corresponds to an internode from the species tree in Fig. 1a. The values on the X axes indicate the median branch length in million years. In **a** and **b**, the Y values are the percentage of gene trees that strongly (**a**) or weakly support (**b**) each given internode. In **c** the Y values represent the site concordance factor (i.e., the percentage of parsimony-informative sites that support each internode). The dashed lines in **b** and **c** represents the predicted values from the linear model we fitted to the log-transformed data. *Indicates that the regression P-value is statistically significant.

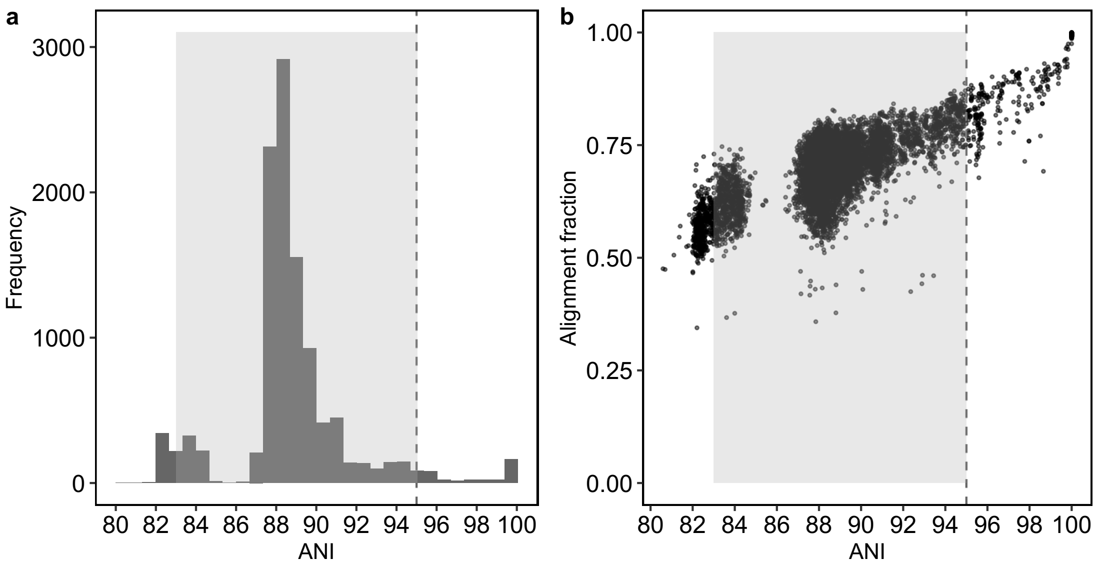


**Supplementary Figure 4.** Distribution of ANI and alignment fraction values between *Nostoc* and *Desmonostoc* genomes shown in Fig. 3. **a** Histogram showing the distribution of all pairwise ANI between all 147 *Nostoc* and *Desmonostoc* genomes included in the study (Supplementary Data 1a). **b** Relationship between genome alignment fraction and ANI. Each dot represents a *Nostoc* genome pair. The gray shade shows the range where the ANI gap is expected. The vertical dashed line shows the 95% ANI threshold typically used for bacterial species delimitation.

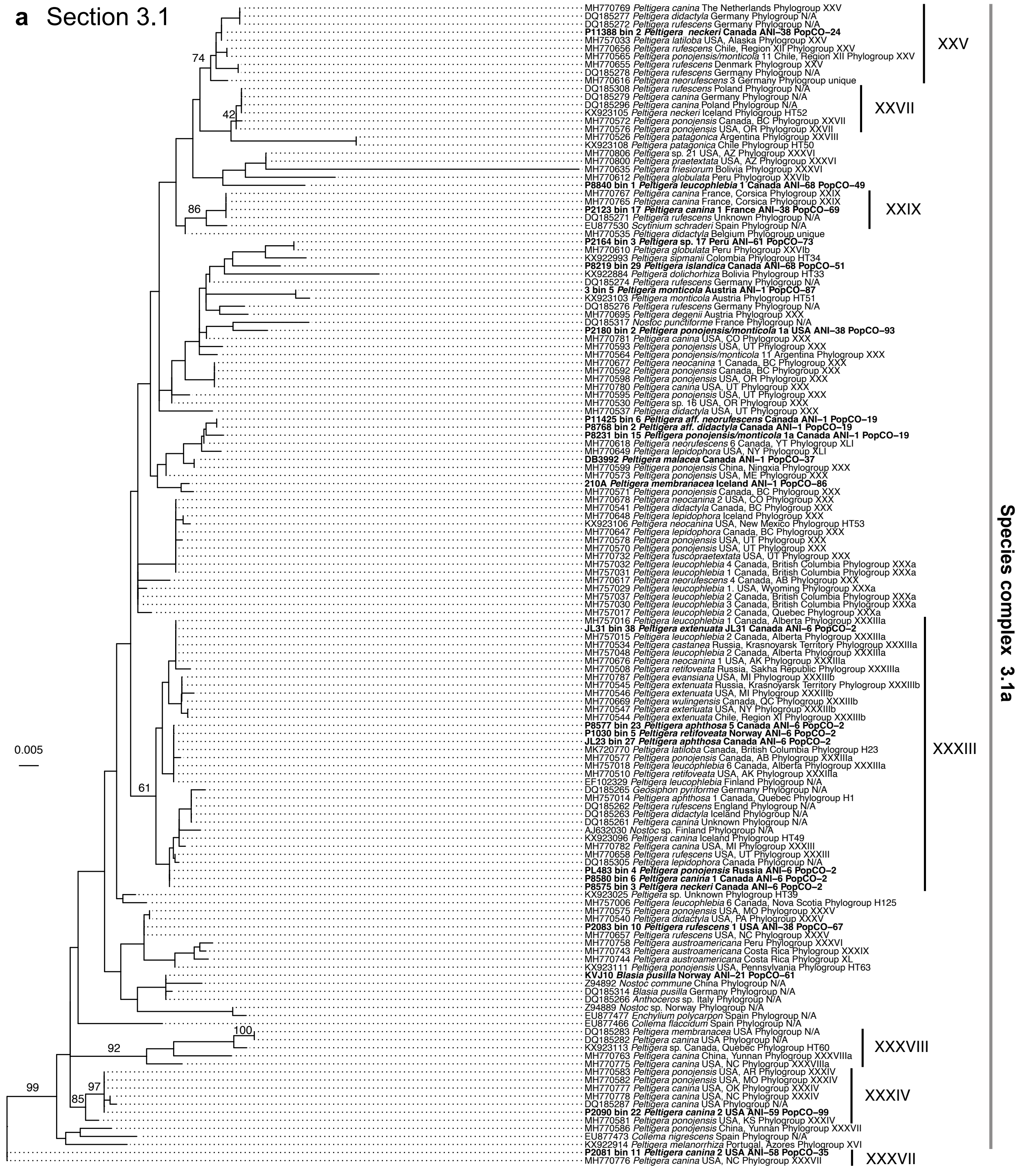


**Supplementary Figure 5.** Phylogenetic trees showing relationships and phylogroup classification within sections of the genus *Nostoc* (as delimited in Figs. 1–3) resulting from maximum likelihood analyses of publicly available and newly generated *rbcLX* sequences. Tip labels for the public sequences include the GenBank accession number, DNA source (i.e., *Nostoc* strain or host name), country of origin, and the original phylogroup designation, if any. Tip labels for the *rbcLX* sequences obtained from *Nostoc*

genomes are shown in bold and include the genome identifier, DNA source, country of origin, ANI 95% cluster number, and PopCOGenT cluster number. Vertical black bars and roman numerals show the *Nostoc* phylogroups that we recognized or validated in this study. Vertical gray bars show the species complexes that we delimited in this study. Numbers above branches are UFBoot2 support values. We only show UFBoot2 values for strongly supported relationships (i.e., UFBoot2 $\geq$ 95%), except when a phylogroup we recognized does not have high support in the *rbcLX* tree. Branch lengths represent expected substitutions per site. **a** Section 3.1. **b** Section 3.2. **c** Section 3.4. **d** Section 3.5. **e** Section 3.6. **f** Section 3.7. **g** Section 3.8. **h** Section 3.9. **i** Section 3.10. **j** Section 3.11. **k** Section 3.12. **l** Section 2.1. **m** Section 2.2. **n** Section 2.4. *Denotes newly circumscribed phylogroups.

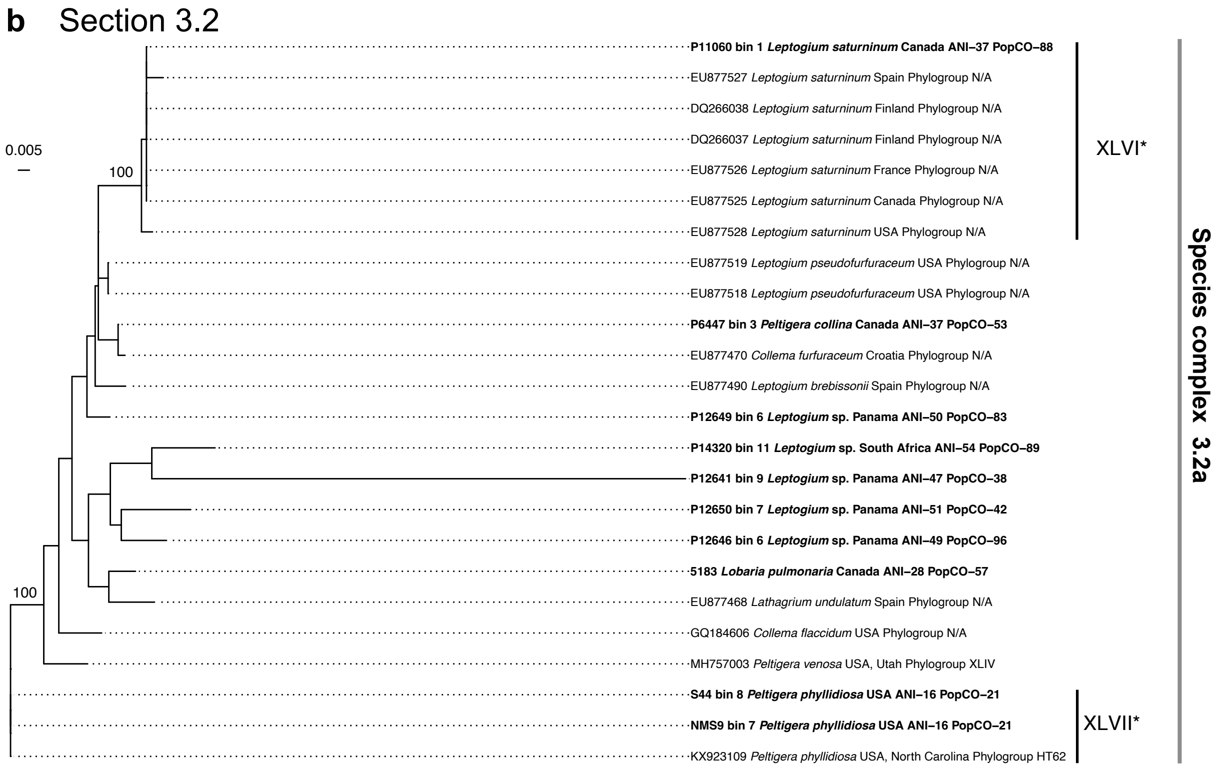


**Supplementary Figure 5** (continued)

## **c** Section 3.4

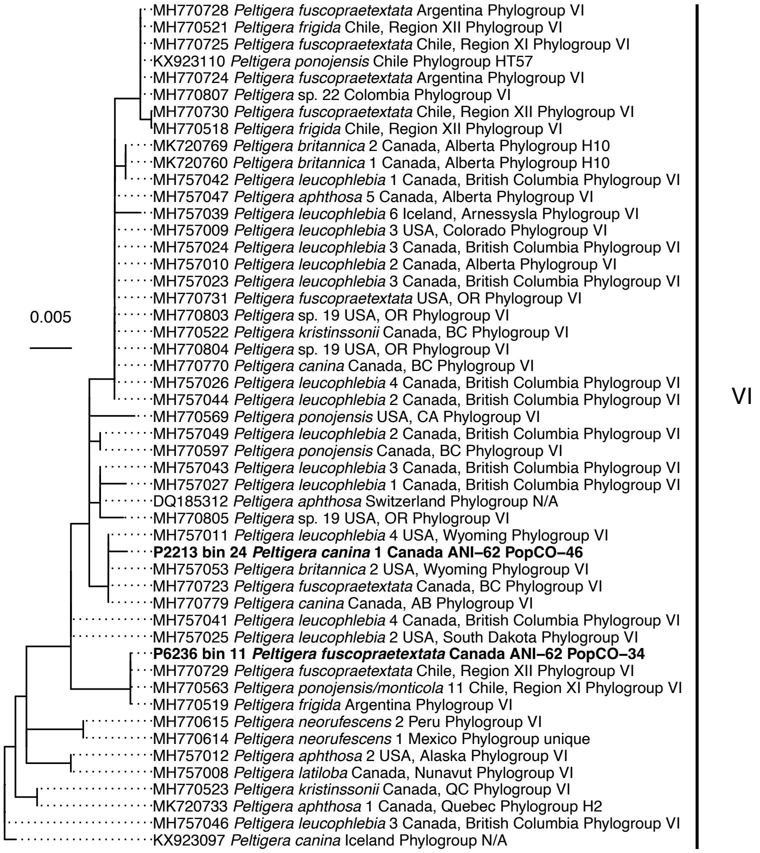


**Supplementary Figure 5** (continued)

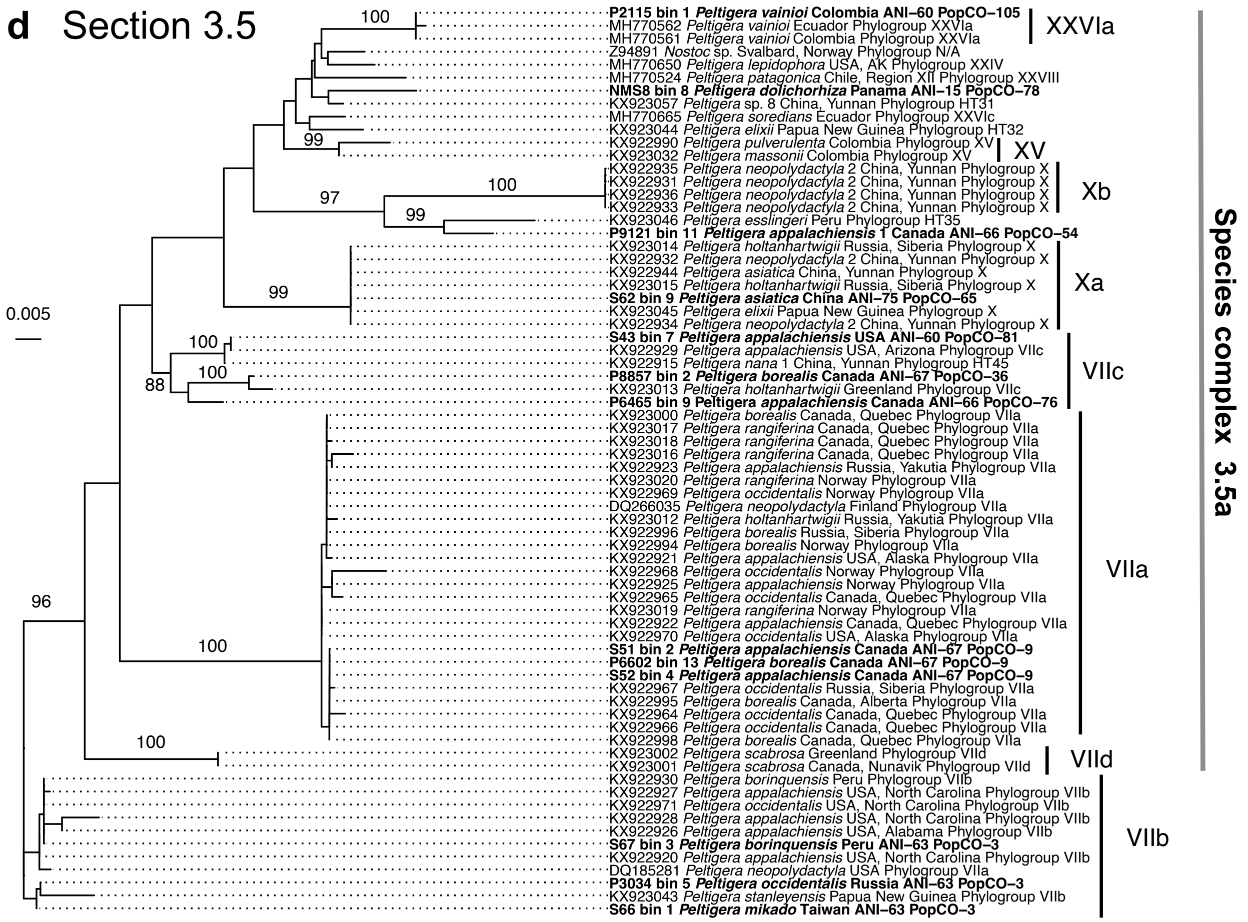


**Supplementary Figure 5** (continued)

**e** Section 3.6

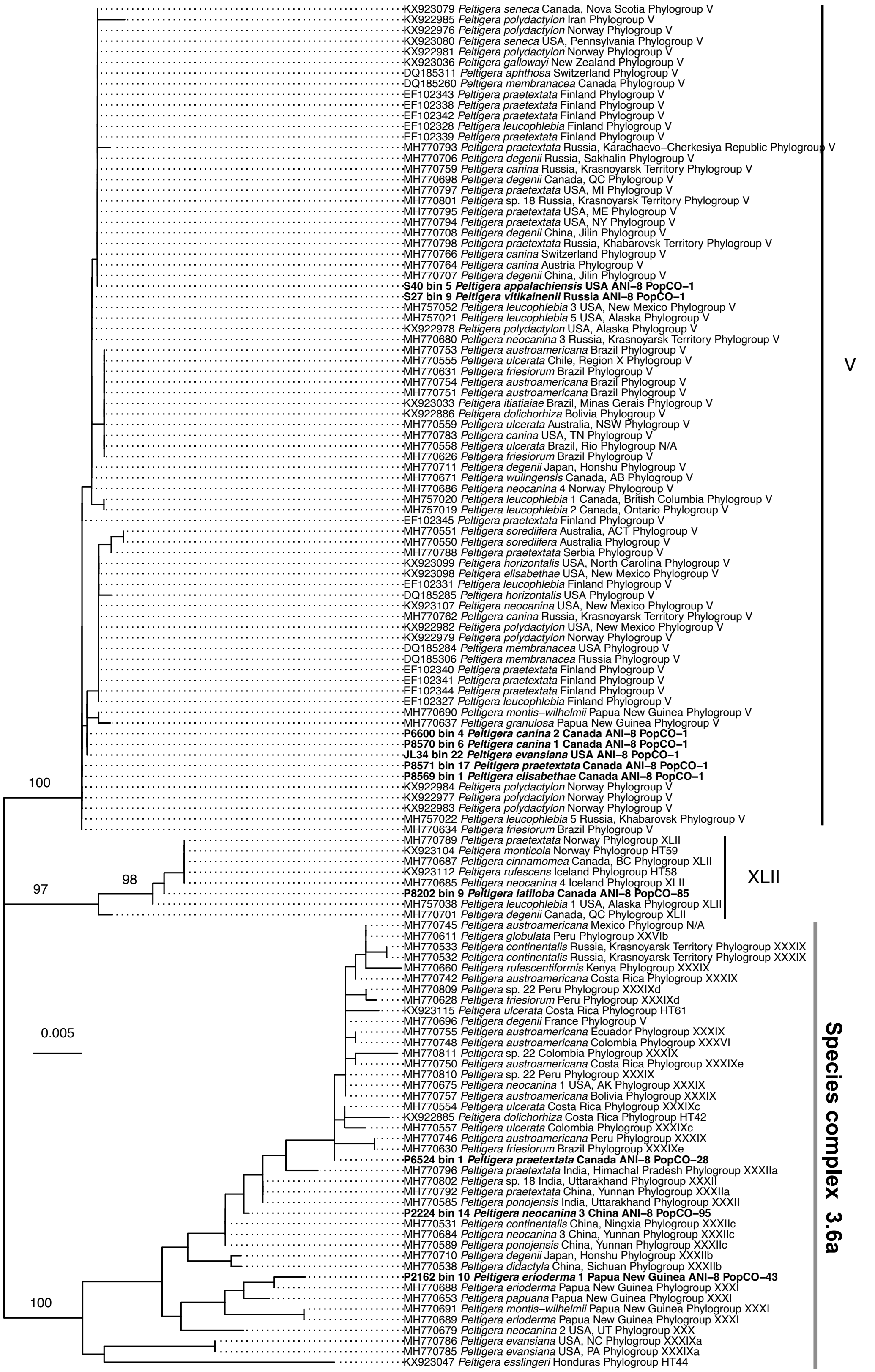


**Supplementary Figure 5** (continued)

**f** Section 3.7

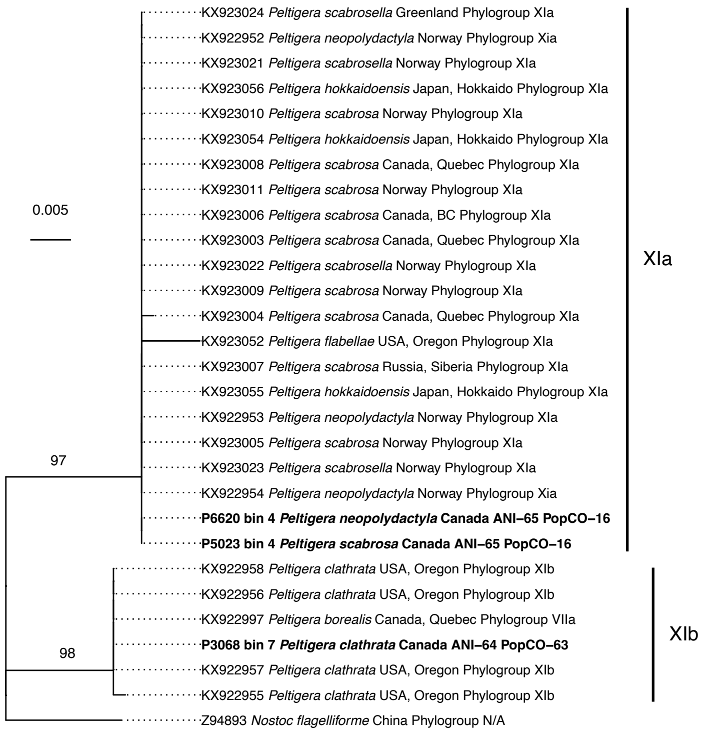


**Supplementary Figure 5** (continued)

**g** Section 3.8

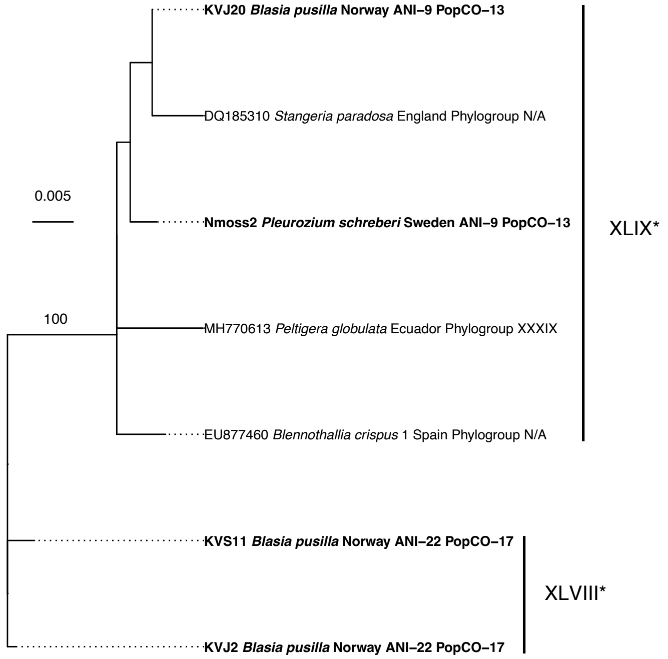


**Supplementary Figure 5** (continued)

**h** Section 3.9

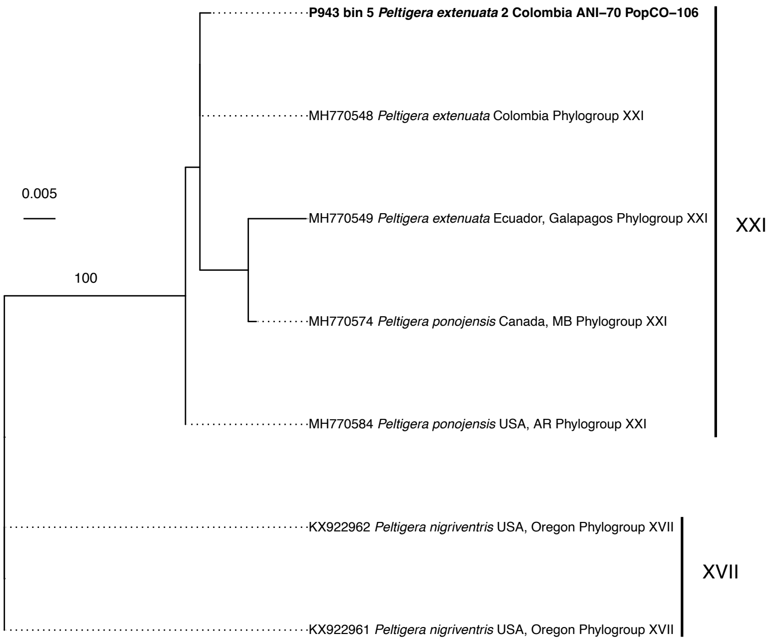


**Supplementary Figure 5** (continued)

**i** Section 3.10

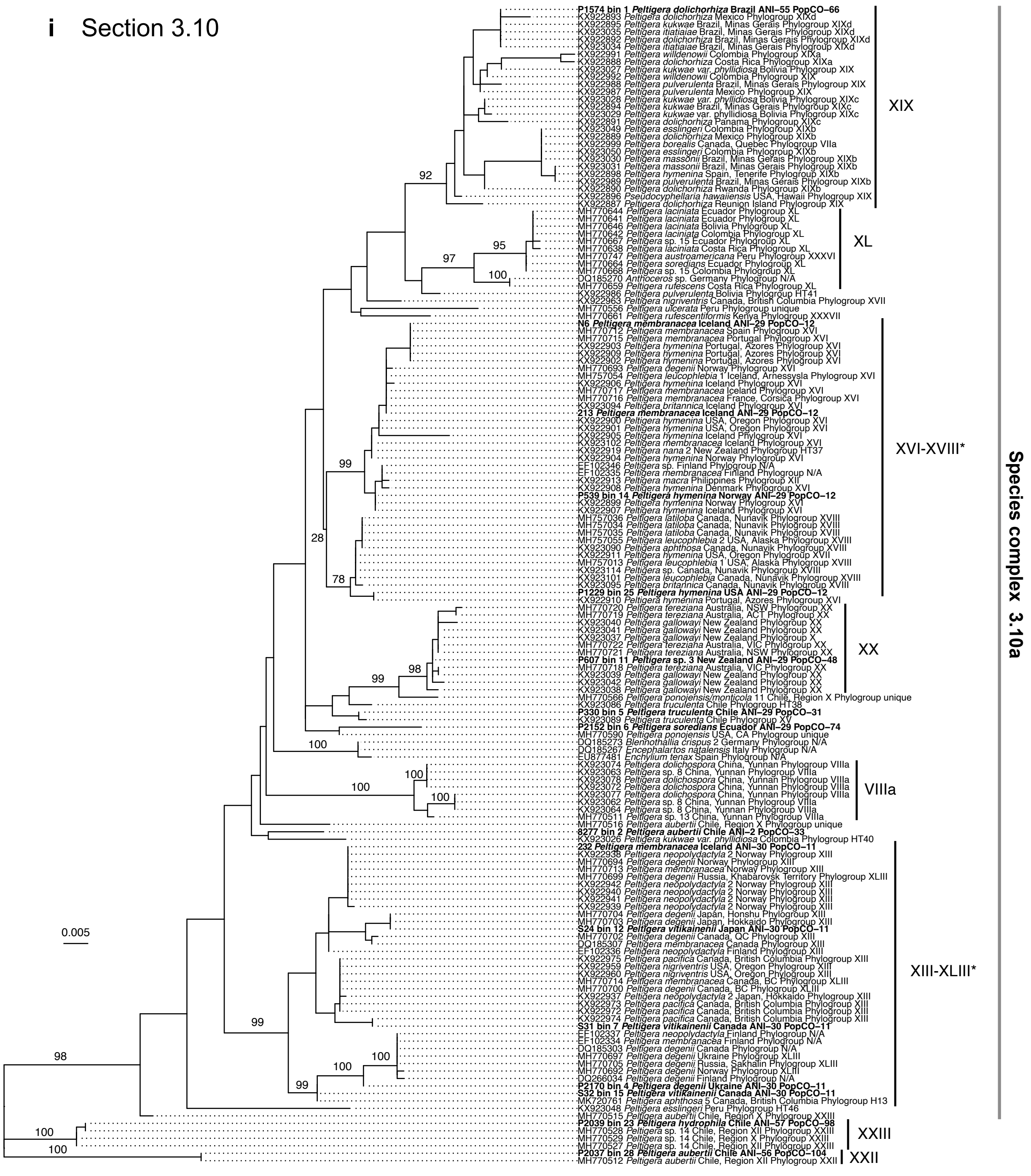


**Supplementary Figure 5** (continued)

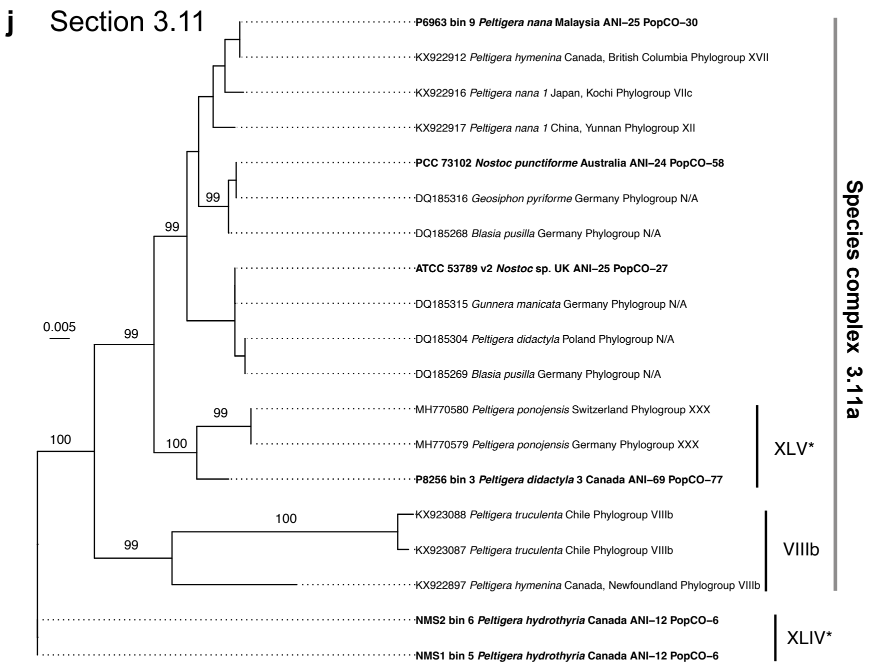


**Supplementary Figure 5** (continued)

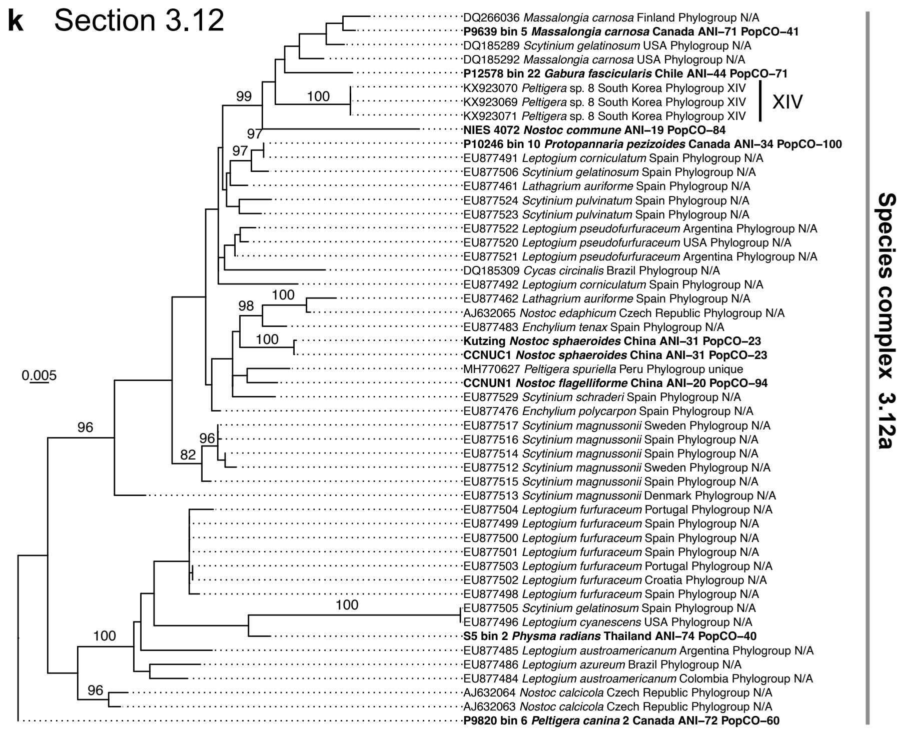


**Supplementary Figure 5** (continued)

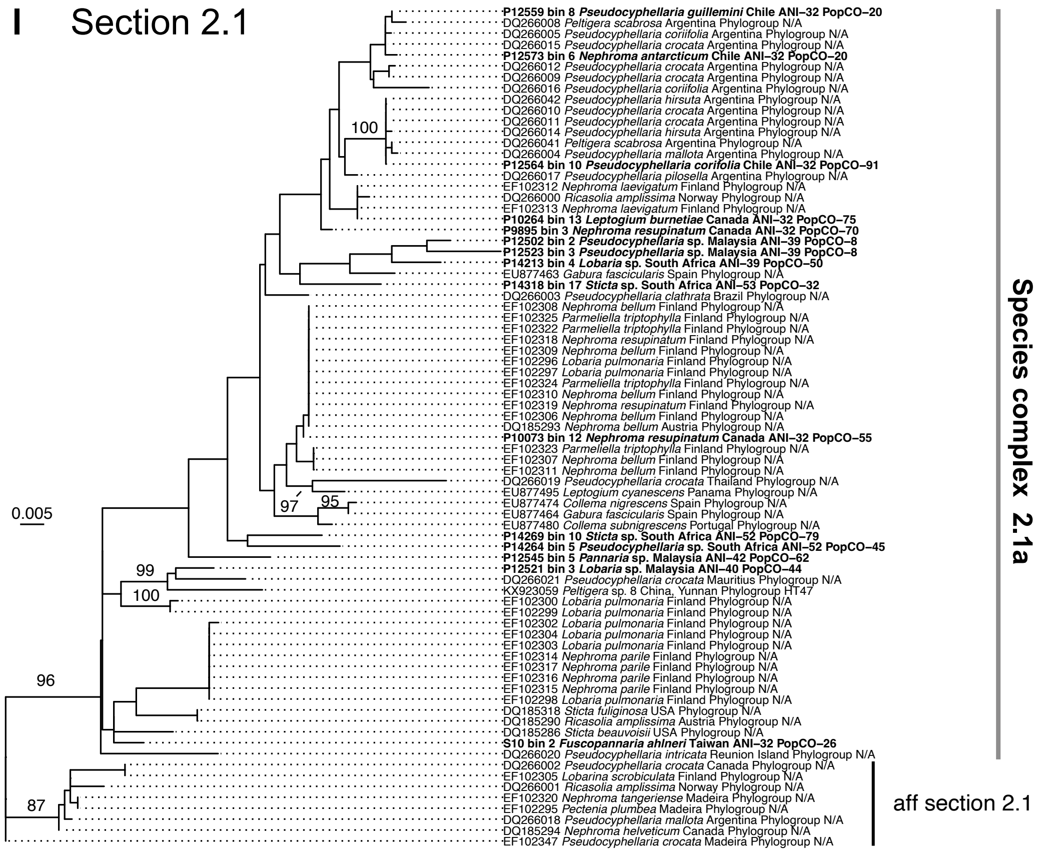


**Supplementary Figure 5** (continued)

**m** Section 2.2

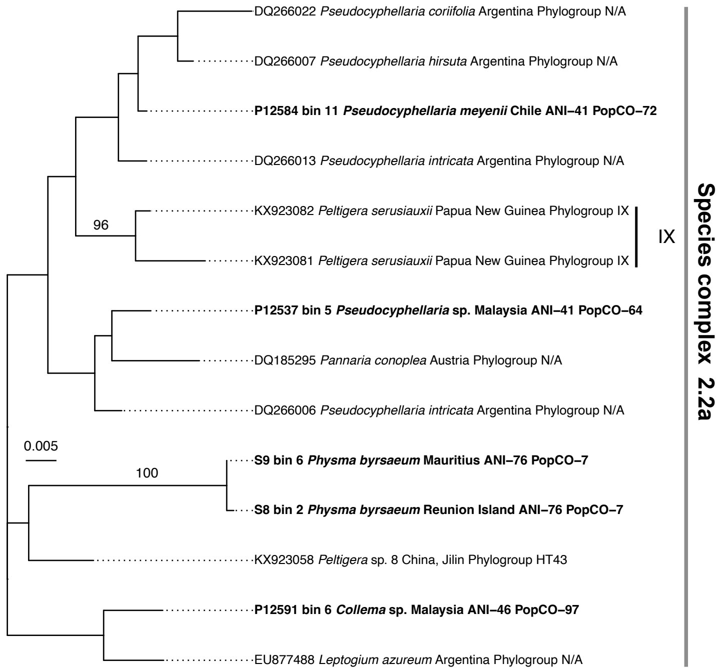


**Supplementary Figure 5** (continued)

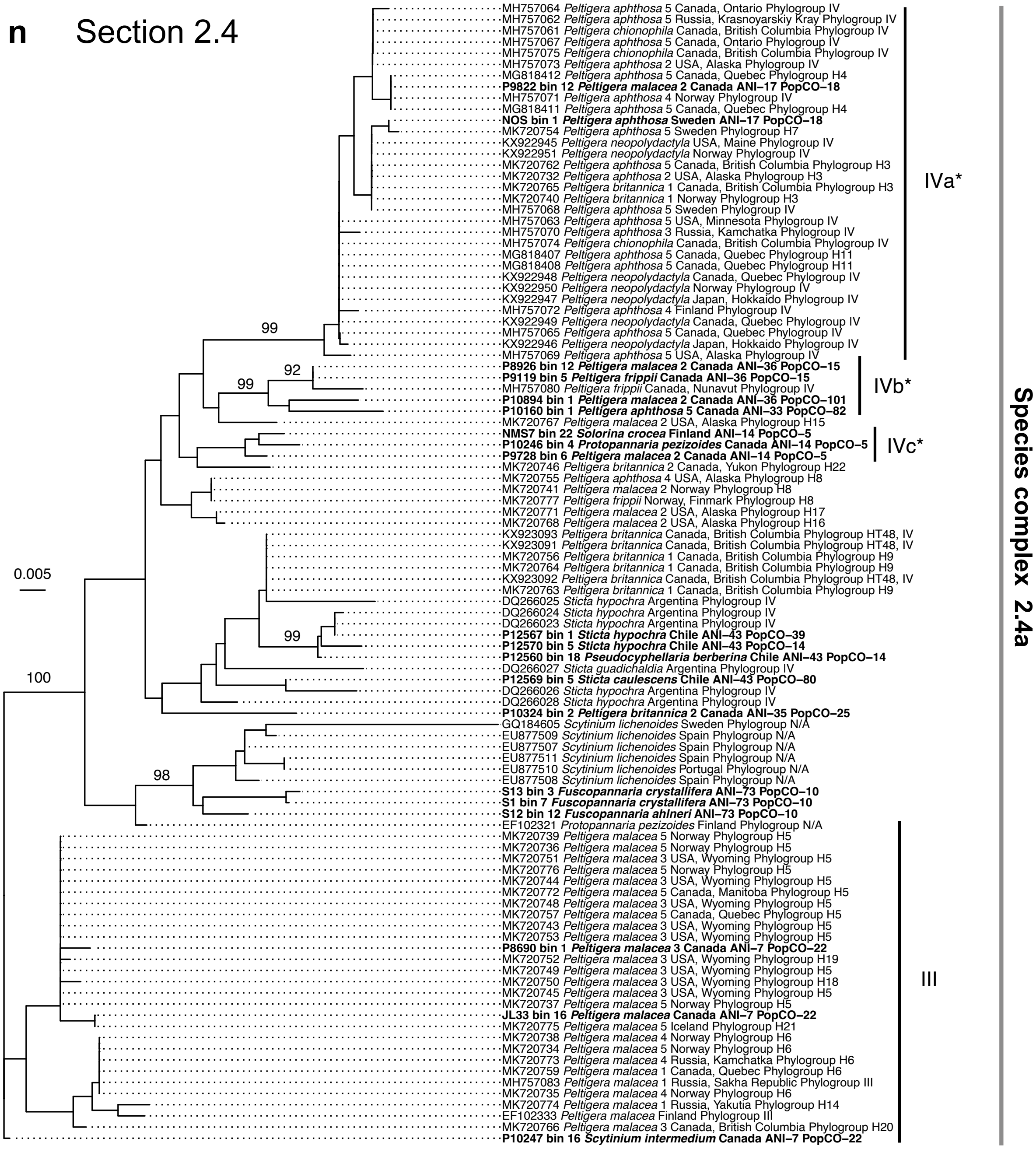


**Supplementary Figure 5** (continued)

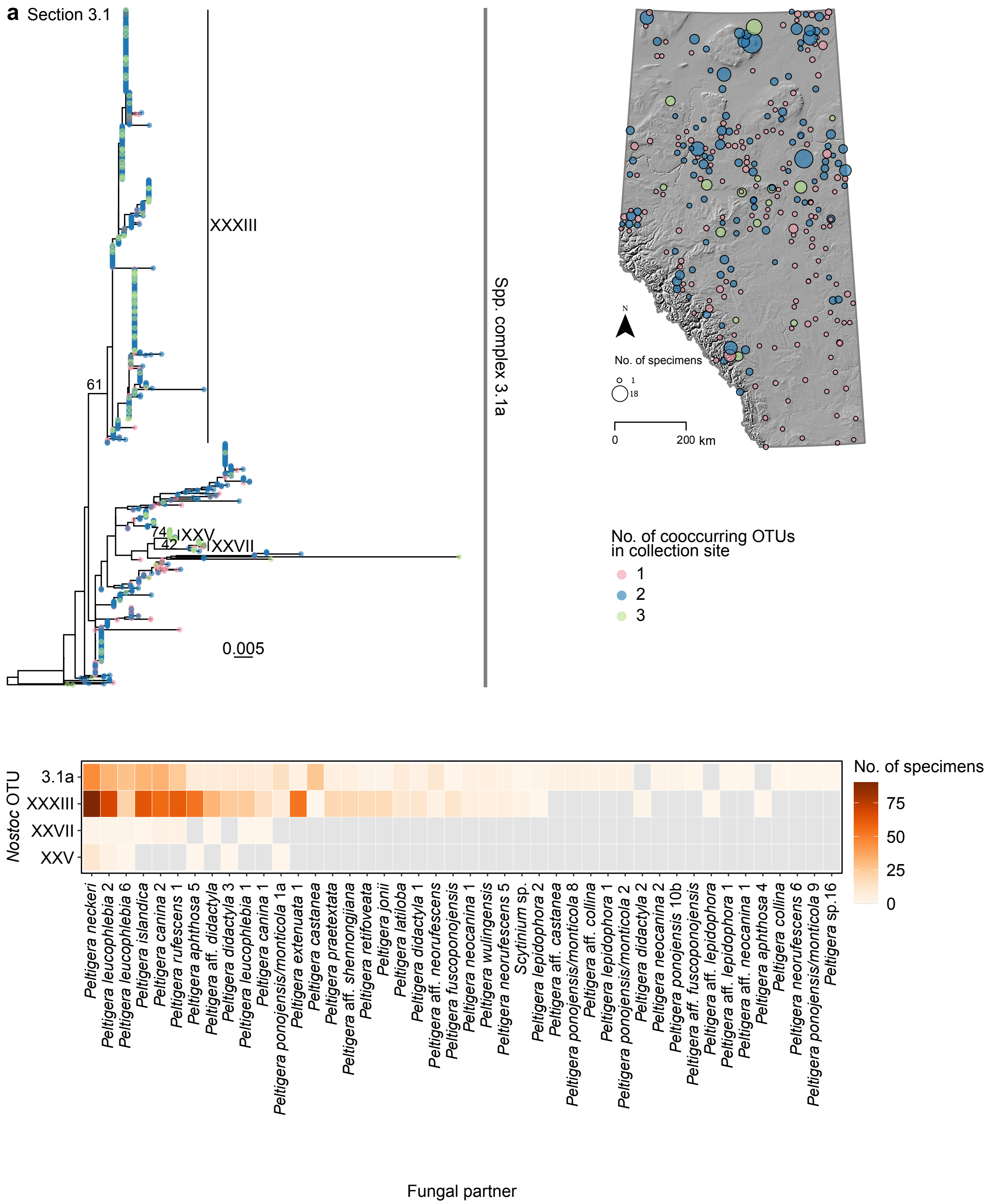


**Supplementary Figure 6.** Additional examples showing lineage boundaries are maintained despite cooccurrence of closely related populations of *Nostoc*. All panels show maximum likelihood trees of *Nostoc rbcLX* sequences of sections (**a**) 3.1, (**b**) 3.5, and (**c**) 2.4 from cyanolichen specimens collected in Alberta, Canada. The color of the

circles at the tips of the tree indicates the number of *Nostoc* OTUs from each section that were present in the site where each lichenized *Nostoc* specimen was collected. We considered each of the phylogroups as one OTU, and all taxa that were not part of a phylogroup were considered as a single OTU (i.e., a species complex). Numbers above branches are UFBoot2 support values. We only show UFBoot2 values for strongly supported relationships (i.e., UFBoot2 $\geq$ 95%), or for branches that define the phylogroups we recognized. Branch lengths represent the expected number of substitutions per site. The relief maps show the distribution of sites where the lichenized *Nostoc* were collected in the province of Alberta, Canada. The heatmaps show the interaction matrices of *Nostoc* strains from each section found in Alberta cyanolichens. Each cell in the matrices shows the number of cyanolichen specimens with a given pair of *Nostoc* OTU and its fungal partner.

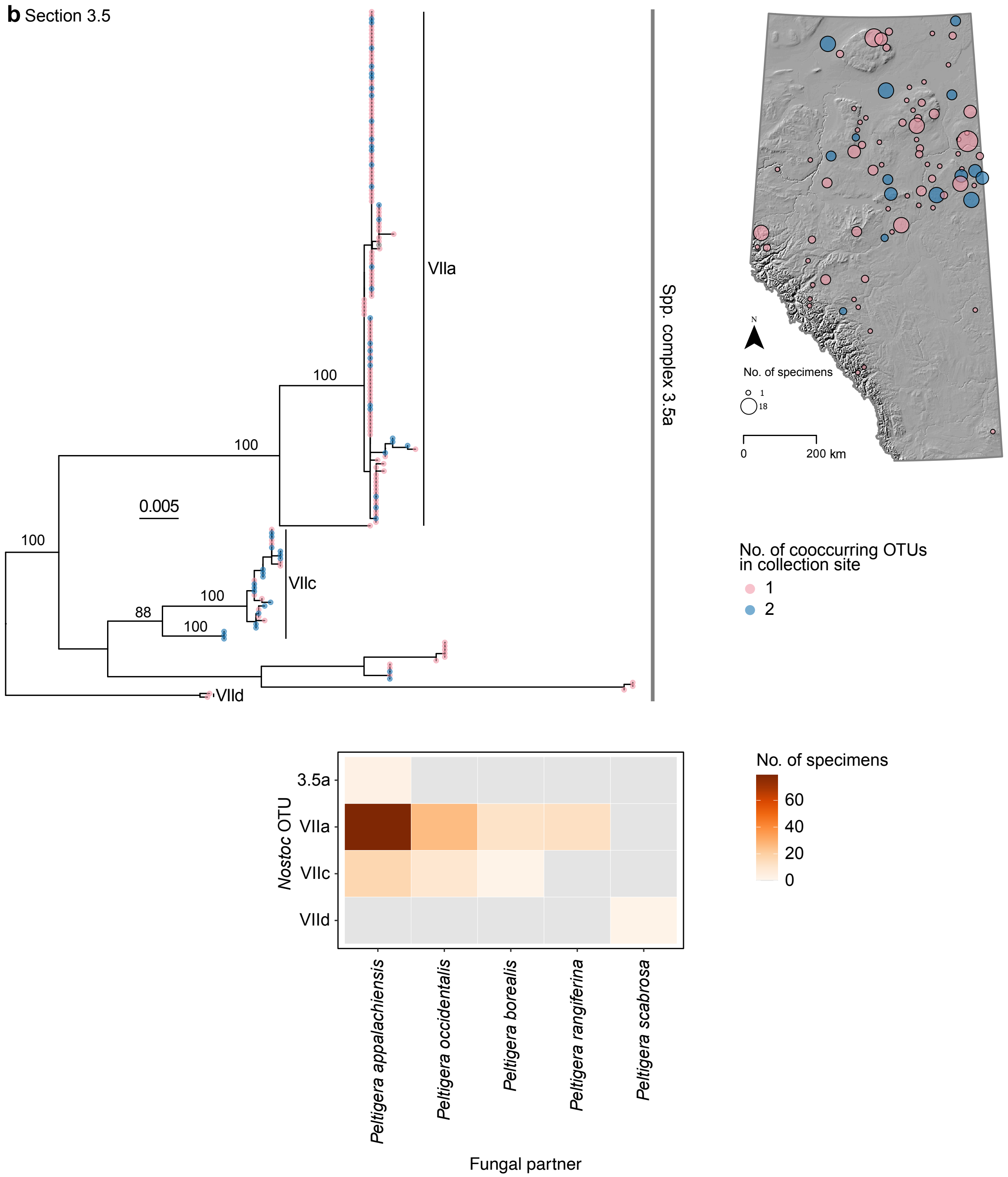


**Supplementary Figure 6** (continued)

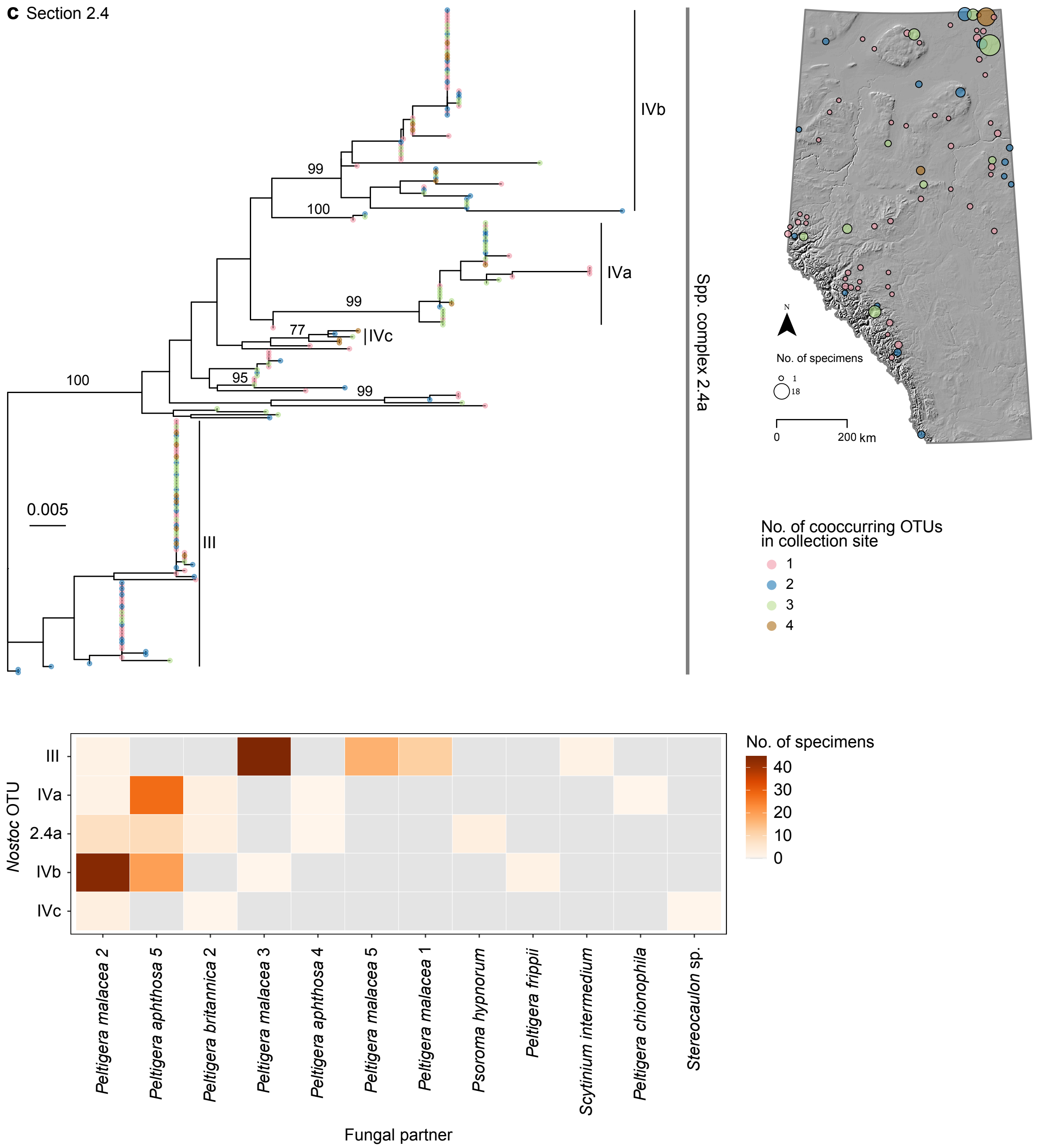


**Supplementary Figure 6** (continued)

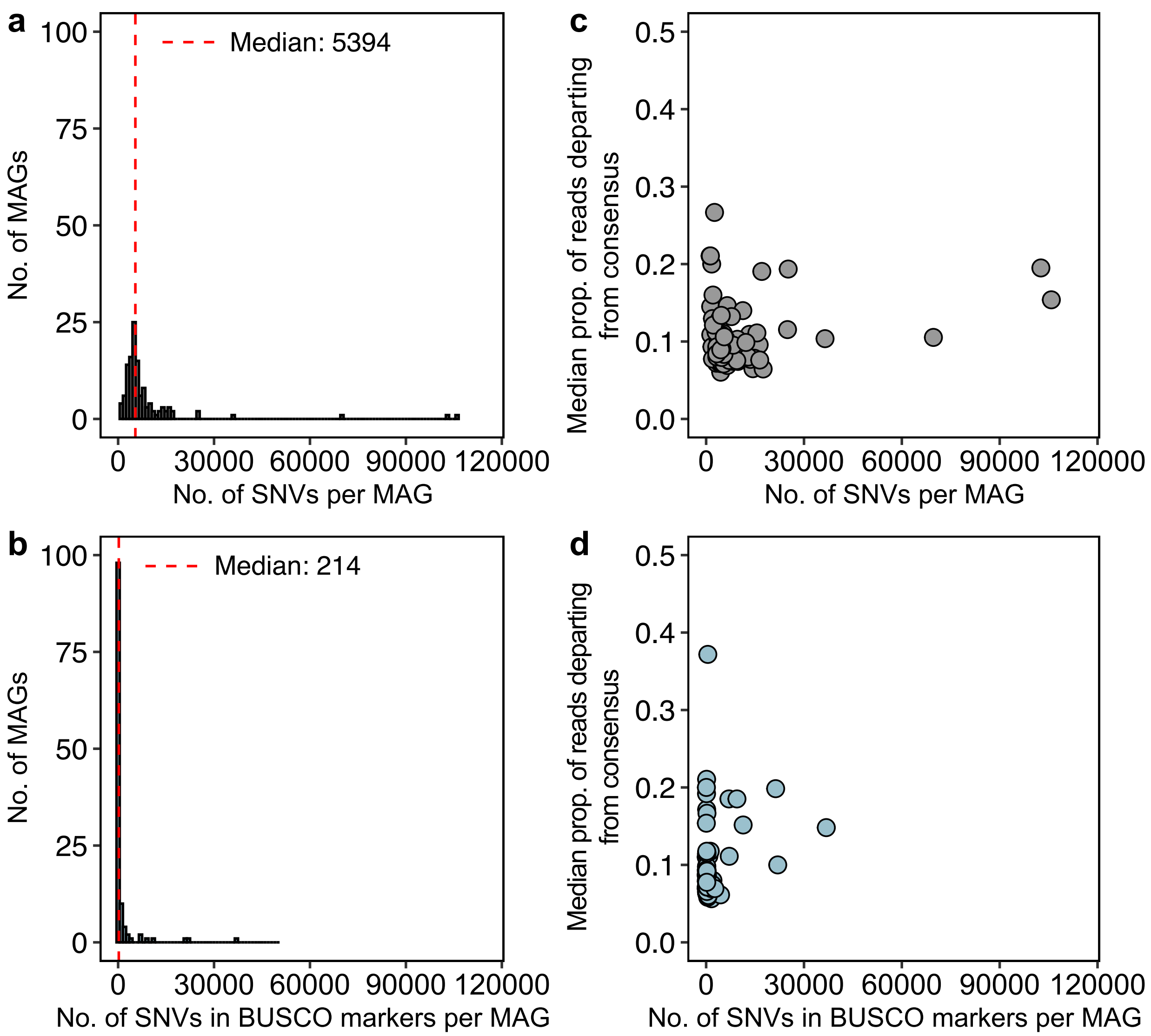


**Supplementary Figure 7.** SNVs are both rare and skewed towards the consensus in *Nostoc* MAGs. **a** Distribution of SNV frequency per MAG. **b** Distribution of SNV frequency within BUSCO markers per MAG. **c** Relationship between SNV frequency per MAG (X axis) and the median proportion of reads that differ from the consensus at each SNV (Y axis). **d** Relationship between SNV frequency within BUSCO marker per MAG (X axis) and the median proportion of reads that differ from the consensus at each SNV within BUSCO markers (Y axis).

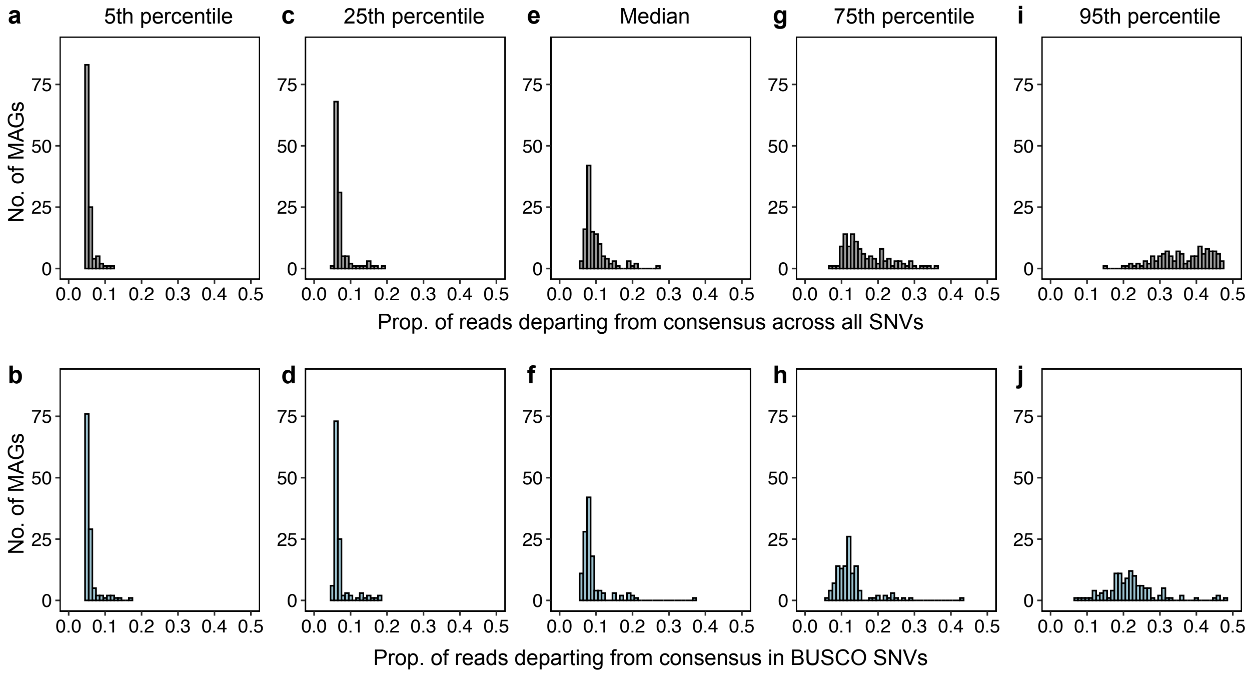


**Supplementary Figure 8.** Summary of the distributions of the proportion of reads that differ from the consensus at each SNV. Each data point in the histograms is a summary statistic of the distribution for each MAG: **a** and **b** 5$^{th}$ percentile; **c** and **d** 25$^{th}$ percentile; **e** and **f** median; **g** and **h** 75$^{th}$ percentile; and **i** and **j** 95$^{th}$ percentile. Dark gray histograms: data from all SNVs across a MAG; blue histograms: data from SNVs within BUSCO markers.

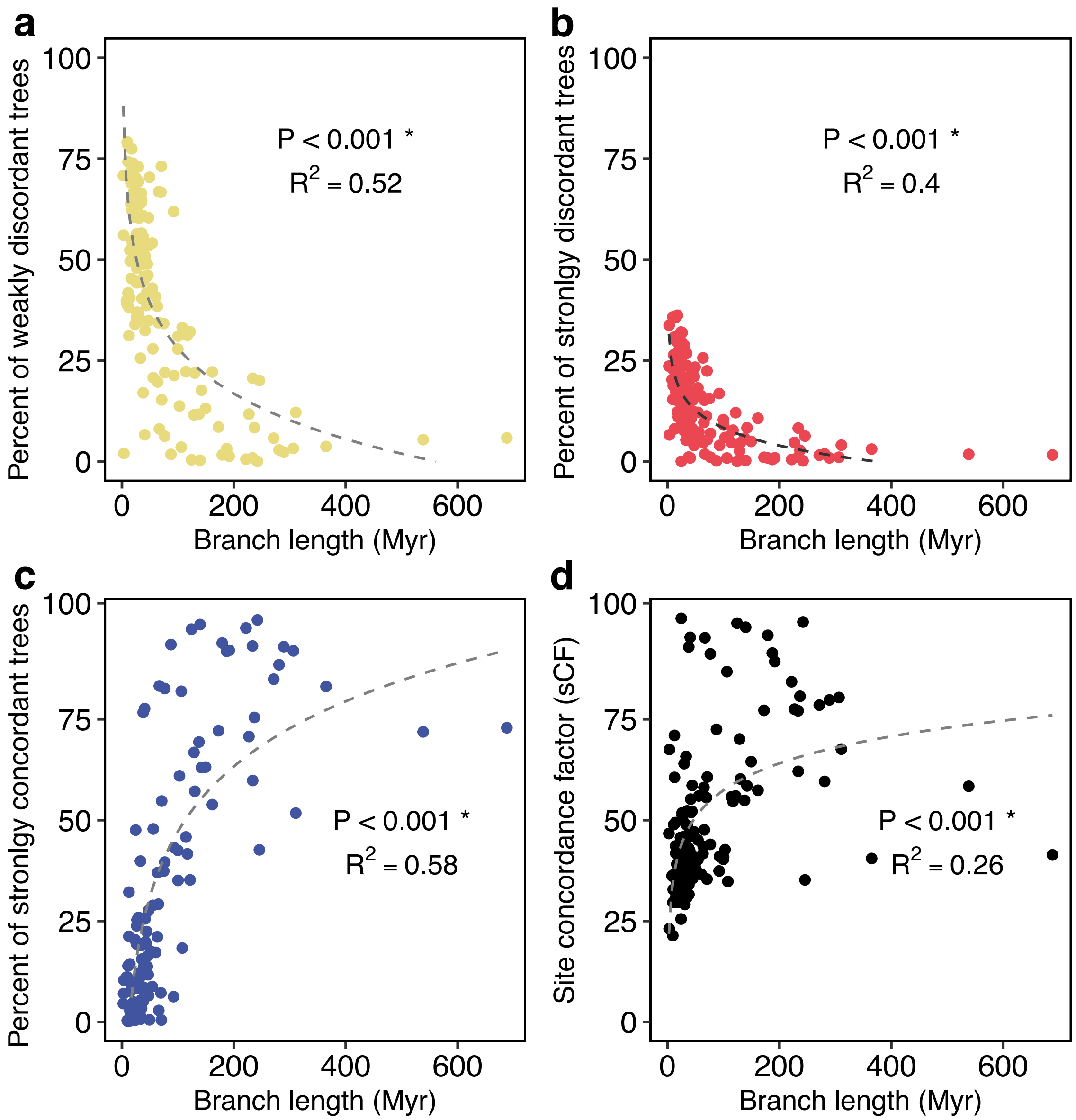


**Supplementary Figure 9.** Patterns of phylogenetic conflict are robust to the removal of loci with SNVs. Each dot corresponds to an internode from the species tree obtained after removing genes with SNVs from the single-locus alignments. The values on the X axes indicate the median branch length in million years. In **a–c**, the Y values are the percentage of gene trees that weakly reject (**a**), strongly reject (**b**), and strongly support (**c**) each given internode. In **d** the Y values represent the site concordance factor (i.e., the percentage of parsimony-informative sites that support each internode). The dashed lines represent the predicted values from the linear model we fitted to the log-transformed data. *Indicates that the regression P-value is statistically significant.
